# T cell migration and effector function differences in familial adenomatous polyposis patients with *APC* gene mutations

**DOI:** 10.3389/fimmu.2023.1163466

**Published:** 2023-07-18

**Authors:** Céline Cuche, Marta Mastrogiovanni, Marie Juzans, Hélène Laude, Marie-Noëlle Ungeheuer, Daniel Krentzel, Maria Isabella Gariboldi, Daniel Scott-Algara, Marianne Madec, Sophie Goyard, Camille Floch, Gaëlle Chauveau-Le Friec, Pierre Lafaye, Charlotte Renaudat, Muriel Le Bidan, Christine Micallef, Sandrine Schmutz, Sébastien Mella, Sophie Novault, Milena Hasan, Darragh Duffy, Vincenzo Di Bartolo, Andrés Alcover

**Affiliations:** ^1^ Institut Pasteur, Université Paris Cité, INSERM-U1224, Unité Biologie Cellulaire des Lymphocytes, Ligue Nationale Contre le Cancer-Équipe Labellisée Ligue 2018, Paris, France; ^2^ Sorbonne Université, Collège Doctoral, Paris, France; ^3^ Institut Pasteur, Université Paris Cité, ICAReB-Clin, Paris, France; ^4^ Institut Pasteur, Université Paris Cité, CNRS-UMR3691, Unité Imagerie et Modélisation, Paris, France; ^5^ Institut Pasteur, Université Paris Cité, Plateforme d’Innovation et de Développement de Tests Diagnostiques, Paris, France; ^6^ Institut Pasteur, Université Paris Cité, CNRS-UMR3528, Plateforme d’Ingénierie des Anticorps, Paris, France; ^7^ Association Polyposes Familiales France, Linas, France; ^8^ Institut Pasteur, Université Paris Cité, Unité de Technologie et Service Cytométrie et Biomarqueurs, Paris, France; ^9^ Institut Pasteur, Université Paris Cité, Hub Bioinformatique et Biostatistique, Paris, France; ^10^ Institut Pasteur, Université Paris Cité, Unité Immunologie Translationnelle, Paris, France

**Keywords:** *Adenomatous polyposis coli*, *APC*, familial adenomatous polyposis, T cell activation, T cell migration, cytokines, immunological synapse, cytoskeleton

## Abstract

Familial adenomatous polyposis (FAP) is an inherited disease characterized by the development of large number of colorectal adenomas with high risk of evolving into colorectal tumors. Mutations of the *Adenomatous polyposis coli (APC)* gene is often at the origin of this disease, as well as of a high percentage of spontaneous colorectal tumors. *APC* is therefore considered a tumor suppressor gene. While the role of *APC* in intestinal epithelium homeostasis is well characterized, its importance in immune responses remains ill defined. Our recent work indicates that the APC protein is involved in various phases of both CD4 and CD8 T cells responses. This prompted us to investigate an array of immune cell features in FAP subjects carrying *APC* mutations. A group of 12 FAP subjects and age and sex-matched healthy controls were studied. We characterized the immune cell repertoire in peripheral blood and the capacity of immune cells to respond *ex vivo* to different stimuli either in whole blood or in purified T cells. A variety of experimental approaches were used, including, pultiparamater flow cytometry, NanosString gene expression profiling, Multiplex and regular ELISA, confocal microscopy and computer-based image analyis methods. We found that the percentage of several T and natural killer (NK) cell populations, the expression of several genes induced upon innate or adaptive immune stimulation and the production of several cytokines and chemokines was different. Moreover, the capacity of T cells to migrate in response to chemokine was consistently altered. Finally, immunological synapses between FAP cytotoxic T cells and tumor target cells were more poorly structured. Our findings of this pilot study suggest that mild but multiple immune cell dysfunctions, together with intestinal epithelial dysplasia in FAP subjects, may facilitate the long-term polyposis and colorectal tumor development. Although at an initial discovery phase due to the limited sample size of this rare disease cohort, our findings open new perspectives to consider immune cell abnormalities into polyposis pathology.

## 1 Introduction

Germline mutations in the *APC* gene are the most common genetic origin of familial adenomatous polyposis (FAP). This inherited disease is characterized by the appearance of hundreds of colorectal and duodenal adenomatous polyps that progress to cancer lesions when left untreated. *APC* gene mutations are also the cause of spontaneous colorectal adenomas and carcinomas (1). Tumor suppressive function of the *APC* gene has been mostly attributed to its role in the Wnt/β-catenin signaling pathway. Thus, APC, together with Axin, scaffold the β-catenin destruction complex, which limits the transcription of β-catenin target genes involved in cell proliferation and differentiation. Reported *APC* mutations may result in the translation of a truncated protein, the lack of translation of the mutated allele or the production of a mutated protein, and consequently, the deregulation of Wnt signaling in various ways (2, 3). However, Wnt-independent APC roles may also contribute to its tumor suppressor function. Indeed, APC is a multifunctional scaffold protein with domains interacting with multiple protein partners. In this way, APC is involved in a variety of cellular processes, including cytoskeleton organization, cell polarity, cell-cell contact, protein stability, RNA localization and processing, among others, which together may influence cell growth and differentiation, polarization, migration, division, and programmed cell death in a variety of tissues (2, 4, 5).

One main function of APC is the regulation of cell polarity. Thus, APC is part of an evolutionarily conserved cell polarity regulatory complex, involving Dlg1, Scribble, atypical Protein Kinase C (aPKC) and Cdc42 (6) Interestingly, APC interacts with the three main cytoskeletal networks, i.e. microtubules, actin and intermediate filaments, controlling their functional interplay during cell migration (7–10). Regulation of cell polarization, adhesion and migration by APC may also contribute to its tumor suppressor function.

The key role of the immune system in tumor control is well established and immunotherapies against numerous types of tumors are now in routine clinical use (11). Various immune cell types are engaged to recognize the presence of the tumor, attract other cells, infiltrate the tumor and eliminate tumor cells. These include dendritic cells, macrophages, NK cells, CD4 helper and regulatory T cells, as well as CD8 cytotoxic T cells. Their functional interplay is crucial for the success of tumor control. Subtle immune regulatory mechanisms are involved improving or impairing an effective anti-tumor immune response (12). Importantly, immune cell infiltration by CD8+ T cells, is a prognostic factor for tumor progression and risk in colorectal cancer (13, 14).

The role of APC in immune cell responses and the potential impact of APC mutations in antitumor immunity remains poorly known. Investigating APC-silenced human T cells and the *Apc^Min/+^* mutant mouse model of polyposis by an array of experimental approaches, we have recently pinpointed several key T cell functions as targets of APC defects (15–17). Thus, APC-silenced human T cells displayed impaired actin and microtubule cytoskeleton organization at the immunological synapse. As a likely consequence, immunological synapses were less polarized, less stable and more irregular in shape and symmetry. In addition, polarized delivery of lytic granules by cytotoxic T lymphocytes (CTL)s was impaired. We also observed defective nuclear translocation of the NFAT transcription factor and reduced production of cytokines. Interestingly, CD4 and CD8 T cells appeared differentially affected by APC defects in some cellular processes and functions, as differentiation and cytokine production, but similarly in others, such as cytoskeleton organization (15, 16). In *Apc^Min/+^* mice, some T cell populations from the intestinal lamina propria, such as regulatory T cells, were altered in terms of numbers and cytokine production, with a stronger effect on the anti-inflammatory cytokine IL10 (15, 18). However, in lymph nodes and spleen no variations in immune cell populations were observed (16). Finally, we recently showed that T cells from FAP subjects carrying APC mutations, or APC-silenced T cells, display impaired T cell adhesion to vascular cell adhesion molecule-1 (VCAM-1) and fibronectin, as well as impaired migration in confined environments (17).

These various findings prompted us to investigate a larger array of immune response features in cells from FAP subjects carrying APC mutations. We used a series of experimental approaches to estimate immune cell responses from stimulated whole blood samples and from purified T cell populations. We compared each FAP subject with an age- and sex-matched healthy subject, processing samples alongside. Our findings indicate differences between FAP subjects and controls concerning several immune response features. These results open new ways to understand the role of immunity in familial polyposis pathology and to envisage future approaches for diagnostics and treatments.

## 2 Subjects, materials and methods

### 2.1 Subjects and blood samples

We recruited FAP subjects with diagnosed mutations in the *APC* gene and the same number of sex- and age-matched (within a 0–5-year difference) healthy subjects through the Clinical Investigation and Access to Biological Resources (ICAReB) Institut Pasteur core facility (NSF 96-900 certified, from sampling to distribution, reference BB-0033-00062/ICAReB platform/Institut Pasteur, Paris, France/BBMRI AO203/1 distribution/access: 2016, May 19th, [BIORESOURCE]). Recruitment and clinical research protocols were approved by a national ethical committee (*Comité de Protection des Personnes, Île de France-1)*. CoSImmGEn cohorts Protocol N° 2010-déc. 12483 for healthy subjects and N° 2018-mai-14852 for FAP subjects). FAP subjects were recruited through the *Association Polyposes Familiales France*. ICAReB ensured all subject visits. All FAP and healthy subjects signed informed consent. Non-inclusion criteria for all subjects were to have a pathology or a treatment potentially affecting immune responses at the time or within the 2 weeks preceding the visit. Each subject filled together with the clinical research physician a health questionnaire. For FAP subjects, this questionnaire included the recording of the mutation type documented by the genetical analysis report extracted from their medical file. A clinical blood analysis was performed at each visit. Subjects were 7 women and 5 men. Average age was 50.4 years for healthy subjects (range 30-69) and 50.1 years for FAP subjects (range 29-72) with an average age matching difference of 2.6 years. APC mutations, according to the genetical diagnosis report, included frameshift mutations potentially leading to lack of protein expression of the mutated allele or expression of truncated forms.

**Figure 1:**
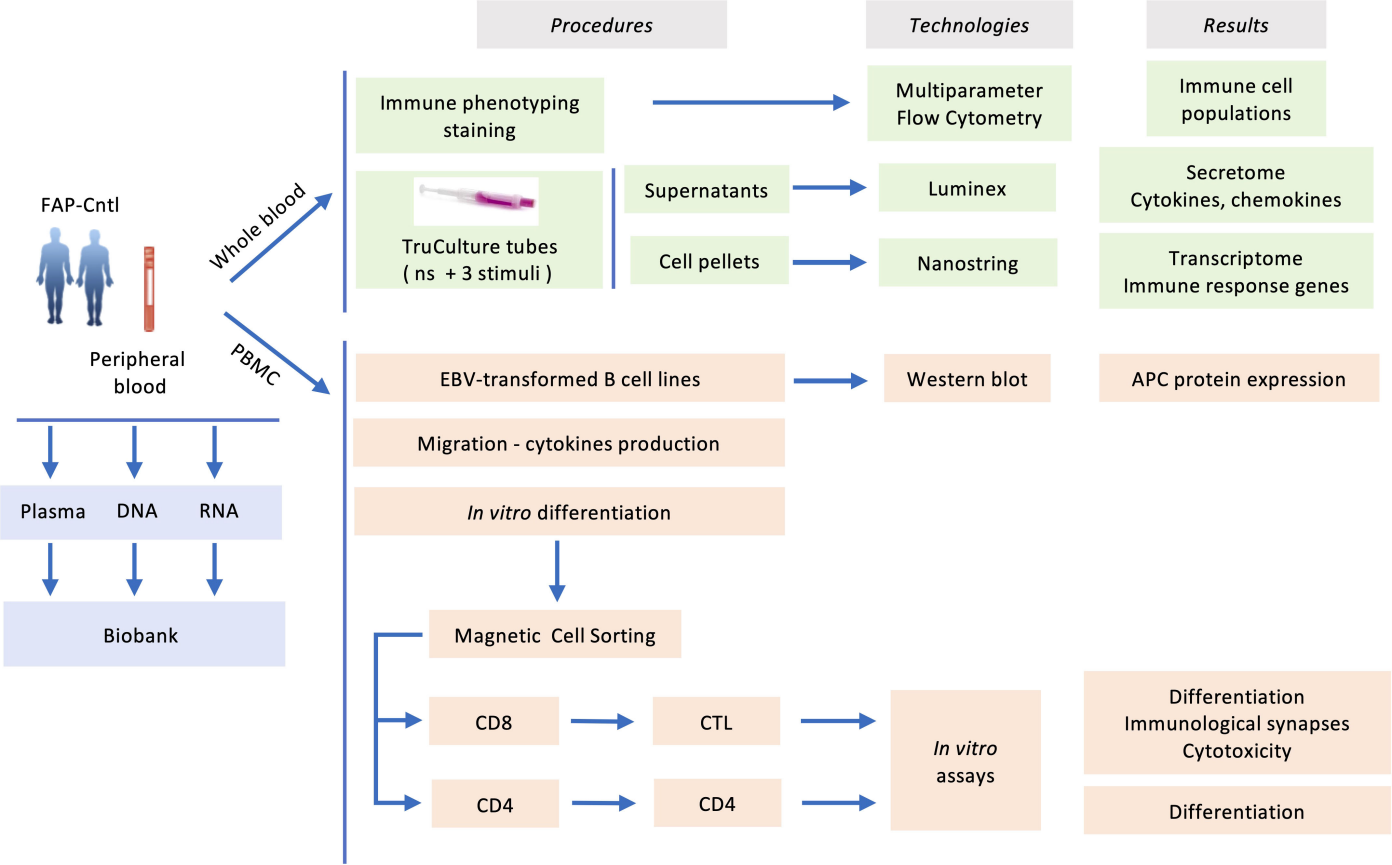
Experimental workflow carried out in the present study.

Blood samples, 100 ml per subject, from both FAP and healthy subjects were withdrawn from the antecubital vein and collected in sodium-heparin tubes. Samples from each pair of FAP and matched healthy subject were collected the same day and analyzed alongside following the workflow depicted in **Figure 1**.

### 2.2 EBV stock preparation

EBV stock was prepared from cultures of the EBV-transformed marmoset cell line B95-8. Cells were cultured at 5 x 10^5^ cells/ml for 12 days to promote the spontaneous reactivation of EBV’s virus release. After 12 days of culture, the cells were centrifuged at 2800 rpm for 15 min at 4°C to separate EBV-containing culture supernatant from cells. Supernatant was passed through a 0.45-micron filter, aliquoted, and stored at -80°C

### 2.3 EBV-transformed lymphoblastoid B cells lines

Lymphoblastoid Cells lines (LCL) were generated by infecting peripheral blood lymphocytes (PBL) with Epstein Barr Virus (EBV). PBMC were adjusted to 5 x 10^5^ cells/ml in RPMI-1640, 20% SVF, 50% freshly thawed EBV supernatant, 0.1 μg/ml cyclosporine and incubated at 37°C, 5% CO_2_ in a 96-well plate. The EBV-infected cells were observed under the microscope to look for rosette-like transformed LCLs in clusters. After having stablished stable cell clones were frozen.

### 2.4 Generation of VHH anti-APC antibodies

A male alpaca was immunized with a protein domain located in the N-terminal of the APC protein (E135 to G279), produced in *E.coli* and purified by affinity chromatography. The sequence of the protein used was as follows (APC amino acids underlined) MEELEKERSLLLADLDKEEKEKDWYYAQLQNLTKRIDSLPLTENFSLQTDMTRRQLEYEARQIRVAMEEQLGTCQDMEKRAQRRIARIQQIEKDILRIRQLLQSQATEAERSSQNKHETGSHDAERQNEGQGVGEINMATSGNGQGLEHHHHHH*.

The immunization process was executed according to the French legislation and in compliance with the European Community Council Directives (2010/63/UE, French Law 2013-118, February 6, 2013). The Animal Experimentation Ethics Committee of Pasteur Institute (CETEA 89) approved this study (2020-27412). Three injections were performed at day 0, 21 and 28, the first one with Freund complete adjuvant and the last 2 ones with incomplete Freund adjuvant. Five days after the third injection, lymphocytes were isolated from an alpaca blood sample. The mRNA was then extracted, and the sequences of the heavy chain antibody variable domains were amplified by PCR. These were inserted into a pHEN 6 phagemid vector. This allowed the construction of the M13 phagemid library exposing the different VHHs on their surface. VHHs specific to the N-terminal fragment of APC were thus selected by the phage display technique, subcloned into a bacterial expression vector pET23d (Novagen) containing a 6-Histidine tag (19). Purified VHHs were isolated by immobilized-metal affinity chromatography (IMAC). VHHs were further ranked according to their affinity for the APC peptide, using LuLISA assays (20). The VHH with the best affinity (in the nanomolar range) was used for the experiments described here.

### 2.5 APC detection in EBV-transformed lymphoblastoid cells lines by immunoprecipitation and western blot

EBV-transformed lymphoblastoid B cells lines were lysed for 15 min in ice-cold buffer containing 0.5% NP40 and a cocktail of protease and phosphate inhibitors (AEBSF 1 mM, Aprotinin 20 μg/ml, Leupeptin 20 μg/ml, chymostatin 50 μg/ml) and phosphate inhibitors (sodium fluoride 10 mM, sodium orthovanadate 2 mM, sodium pyrophosphate 10 mM, Beta glycerol-phosphate 40 mM) and insoluble material was removed by centrifugation at 20800 x *g* for 10 min at 4°C. Immunoprecipitations were performed by incubating lysates for 1.5–2h at 4°C with Streptavidine–coupled Sepharose beads (Iba) previously coated with biotin-labelled VHH anti-APC antibodies. Beads were then washed 3 times and bound proteins were eluted by heating them at 70°C in sample loading buffer. Protein electrophoresis was performed on NuPAGE gels (3-8% of acrylamide) (Invitrogen). After protein electrotransfer on nitrocellulose membrane (LI-COR Biosciences), immunoblots were saturated with blocking buffer for near-infrared fluorescent western blotting (Rockland Immunochemicals) and incubated with an anti-APC primary antibody (clone Ali 12-28; 2 μg/ml; Abcam). After membrane washing and incubation with secondary antibodies Alexa Fluor 680 (Invitrogen), near-infrared fluorescence was detected by using an Odyssey CLx scanner and the Image Studio v5.2 software (LI-COR Biosciences).

### 2.6 Whole blood cell immunophenotyping

Three ml of whole blood were washed by mixing fresh whole blood and PBS at a 1:9 ratio, followed by centrifugation at 200 xg for 5 min at room temperature (18-22°C). Cell pellets were resuspended in 9 ml of BD Pharm Lyse™ 1x (BD Biosciences), shortly vortexed and incubated 20 min at room temperature protected from light under mild agitation. Samples were washed twice with PBS at a ratio 1:9, followed by centrifugation at 200 xg for 5 min at room temperature. Cell pellets were incubated 25 min in the dark at room temperature with 1x viability dye, washed twice with PBS + 0.5% BSA, then centrifuged at 500 xg for 5 min at room temperature. Cell pellets were resuspended with PBS+0.5% BSA. The equivalent of 300 μl of whole blood was split into 6 tubes containing 6 different premixed antibody cocktails (see below), incubated 25 min in the dark at room temperature, washed twice with PBS + 0.5% BSA and centrifuged at 500 xg for 5 min at room temperature. Cells were resuspended and fixed with PBS + 0.5% BSA + 1% PFA.

Thirty-seven different cell surface molecules defining different immune cell lineages and populations were identified using monoclonal antibodies directly labelled with an array of fluorescent dyes. Antibodies and fluorochromes are listed in **Supplementary Table 1**.

Labelled cells were analyzed the following day by multiparameter flow cytometry using a Spectral Flow Cytometer SP6800 or ID7000 (Sony). Data analyses were performed with FlowJo software. Gating strategies for the different panels are shown in **Supplementary Figures 1-3**.

### 2.7 Whole blood immune cell activation in TruCulture^®^ tubes

Standardized TruCulture^®^ tubes (RBM) were purchased pre-filled with a proprietary medium alone (null control) or supplemented with either anti-CD3 (clone UCHT1; 0.4 μg/ml) plus anti-CD28 (clone CD28.2; 0.33 μg/ml), *Staphylococcus Enterotoxin* B superantigen (SEB; 0.4 μg/ml) or heat killed *Candida albicans* (HKCA; 10^7^ fungi/tube) and stored frozen at -20°C. The day of the experiments, tubes were thawed at room temperature for 1 h before use. 1 ml of whole blood was drawn into each tube within 15 min after sampling, then tubes were transferred to a heat block and incubated at 37°C for 22 h ± 15min. Then, tubes were opened, and the valve separator was inserted *via* a plunger to separate cells and supernatant. The supernatant was transferred to microfuge tubes and stored at −80°C and the cells were transferred to Trizol for RNA extraction and stored at -80°C (21).

### 2.8 RNA extraction and quality control

Cell pellets in Trizol LS were thawed on ice 60 min prior to processing. To complete thawing and RNA release, tubes were vortexed twice for 5 min at 2x10^3^rpm. Before processing, thawed samples were centrifuged at 3x10^3^ xg for 5 min at 4°C to pellet the cellular debris generated during the Trizol lysis. For extraction, we use the NucleoSpin RNA kit (Macherey-Nagel). In brief, 900 μl of the clarified phase of the Trizol lysate was transferred to a tube preloaded with 900 μl of 100% ethanol. The binding mixture was transferred into the silica membrane tube. The columns were washed with buffers RAW2 and RA3 and RNA eluted into a 1.5 ml nuclease-free collection tube (Macherey-Nagel) using 40 μl RNase-free water. To avoid unnecessary freeze and thaw of the RNA, distinct aliquots for quality control, quantification and gene expression analysis were prepared, and all aliquots were frozen at -80°C until use. RNA concentration was estimated using Qubit RNA HS Assay Kit (Life Technologies) according to the protocol provided by the manufacturer. An automated RNA integrity assessment was performed using the RNA Pico Chips Kit on the Agilent 2100 Bioanalyzer System (Agilent Technologies). The RNA integrity number (RIN) was calculated using the Agilent 2100 Bioanalyzer System software, and all samples with a RIN greater than four were processed for gene expression analysis.

### 2.9 Gene expression by NanoString technology

Total mRNA was diluted with RNase-free water at 20 ng/ml in the 12-strip provided by NanoString. We analyzed 100 ng of total RNA from each sample using the Human Immunology kit v2 according to manufacturer’s instructions. Each sample was analyzed in a separate multiplexed reaction including in each, eight negative probes and six serial concentrations of positive control probes. Nanostring nCounter Immunology V2 panel (Cat No XT-CSO-HIM2-12) analyzing 579 immune response genes was used (**Supplementary Table 2**). Negative control analysis was performed to determine the background for each sample. Data was imported into nSolver analysis software (version 4.0) for quality checking and normalization of data. Several subjects did not respond to anti-CD3+anti-CD28 stimuli, as previously described (21). Their data were not considered for further analysis.

### 2.10 Immune secretome by Luminex technology

Supernatants from whole blood stimulated in TruCulture^®^ tubes were analyzed by Luminex xMAP technology, using a multiplex kit measuring 44 different analytes (**Supplementary Table 3**) (Luminex Performance Assay, Human XL Cytokine Fixed Panel, ref LKTM014, R&Systems). TruCulture^®^ supernatants were tested neat and at 1/3 dilutions. Secreted amounts were normalized with respect to non-stimulated samples. As for the Nanostring analysis, data from non-responders under CD3/CD28 stimulation were not considered for further analysis.

### 2.11 PBMC isolation and activation

Peripheral blood mononuclear cells (PBMC) were purified from approximately 80 ml of blood from FAP or healthy subjects by Ficoll-Hypaque centrifugation (Lymphoprep 114-545 AbCys) and activated with TransAct™ (1:100; Miltenyi-Biotec) and recombinant human IL-2 (100 IU/ml; PeproTech) in RPMI-1640 medium supplemented with GlutaMAX-I (Gibco), 5% human serum, 1 mM sodium pyruvate, nonessential amino acids, 10 mM HEPES, 1% penicillin-streptomycin (v/v).

### 2.12 Purification of CD4^+^ and CD8^+^ T cells

CD4 and CD8 T cells were purified from a fraction of PBMC (≈15x10^6^) at 5 days of activation. CD4 T cells were isolated using positive magnetic sorting (Miltenyi-Biotec) and CD8 T cells recovered from the non-retained population.

### 2.13 *Ex vivo* differentiation of peripheral blood T cells

PBMC were purified as indicated above and activated with TransAct™ (1:100, Miltenyi-Biotec 130-111-160) and recombinant human IL2 (100 IU/ml; PeproTech). Then at days 0 and days 2-4-6 post-stimulation, the cells were stained with Fixable Viability Stain 450 (250 ng/mL; BD Biosciences), next with anti-CD3-PE-Cy5 (1/30; clone HIT3a; BD Biosciences), anti-CD4-PE-Vio770 (1/100; clone rea623; Miltenyi Biotec), anti-CD8-APC-Cy7 (1/30; clone RPA-T8; Biolegend), anti-CD25-Alexa fluor 488 (1/30; clone M-A251; Biolegend), anti-CD45RA-APC (1/30; clone HI100; Biolegend), anti-CCR7-PE (1/50; clone G043H7; Biolegend), anti-Granzyme-B-PE (1/50; clone GB12; Invitrogen), anti-PD1-Alexa 488 (1/50; clone EH12-2H7; Biolegend) and anti-KLRG1-APC (1/100; clone Rea261; Miltenyi Biotec). FACS analyses were performed on a MACSQuant Analyzer (Miltenyi Biotec) and data analyzed using FlowJo 10 software (FlowJo, LLC). All samples were gated on forward and side scatter, for singlets and for live cells.

### 2.14 Detection of cytokine production *ex vivo* by peripheral blood T cells at day 0 and day 7

At day 0 or day 7 post stimulation performed as above, 4x10^5^ PBMC or CTLs were washed and stimulated or not in a 96-well plate pre-coated with anti-mouse IgG (4 μg/ml; Southern Biotech) followed by anti-CD3 antibodies (5 μg/ml; UCHT1, Biolegend) and soluble anti-CD28 antibodies (2 μg/ml; Beckman Coulter) or (Phorbol 12-myristate 13-acetate (PMA) (50 ng/ml) and Ionomycin (1 μg/ml). Two hours later, brefeldin A (10 μg/ml, Sigma-Aldrich) was added. At 6 hours of stimulation, cells were fixed with 4% PFA for 15 min, at room temperature, permeabilized in PBS 1X BSA 0,5%, saponine à 0,05% for 15 min at room temperature and subsequently stained with anti-IL-2-PerCP-Cy5.5 (1/50; clone MQ1-17H12; BD Biosciences), anti-TNF-FITC (1/100; clone cA2; Miltenyi biotech), anti-IFNγ-Alexa fluor 647 (1/50; clone 4S.B3; BD Biosciences) and only for day 7 anti-Granzyme B-PE (1/50; clone GB12; Molecular Probes), in the presence of 0.05% saponin. FACS analyses were performed as above. Similar experiments were performed at day 7, after removing IL2.

### 2.15 T cell proliferation

PBMCs (4x10^5^) from FAP and healthy subjects were stimulated in a 96-well plate coated sequentially with anti-mouse IgG (4 μg/ml; Southern Biotech), then with anti-CD3 antibodies (5 μg/ml; UCHT1, Biolegend). Soluble anti-CD28 antibody (2 μg/ml; Beckman Coulter) was added to the medium and cells were incubated for 72 h at 37°C. In indicated experiments, PBMCs were labeled with 5 μM of CellTrace™ violet cell proliferation kit (Invitrogen) previous to stimulation, for assessment of cell proliferation. Three days post stimulation, cells were stained with Zombie NIR™ Fixable viability kit (1/1000; Biolegend), next with anti-CD3-APC (1/40; clone UCHT-1; eBiosciences), anti-CD4-PE-Vio770 (1/100; clone rea623; Miltenyi Biotec), anti-CD8-PE (1/10; clone Leu-2a; Beckman Coulter). FACS analyses were performed as above.

### 2.16 Degranulation assay

CTLs obtained from PBMC at day 7 post stimulation and day 2 after purification were mixed at a 1:1 ratio with P815 target cells previously stained with cell proliferation dye efluor 670 (1/2000; ebiosciences) and coated with indicated concentration of anti-CD3 (OKT3 produced by A. Alcover) in the presence of anti-CD107a-PE (1/90, clone H4A3, Biolegend) and incubated for 3h at 37°C. Cells were stained with anti-CD8a-APC-Cy7 (1/30; clone HI100; Biolegend). Facs analyses were performed as above.

### 2.17 Cytotoxicity assay

CTLs obtained from PBMC at day 7 post stimulation and day 2 after purification were resuspended in RPMI 1640 + GlutaMAX-I (Gibco) 3% FCS, 10 mM HEPES, and mixed at the indicated CTL:target cell ratio with P815 target cells previously coated with anti-CD3 (6 μg/ml; UCHT1, Biolegend) for 45min at 4°C. Mixed CTLs and target cells were incubated for 4h at 37°C. The percentage of target cell lysis was measured using the CytoTox-96^®^ Non-Radioactive Cytotoxicity Assay (Promega) following manufacturer’s instructions. Absorbance at 490 nm was measured using a PR2100 Microplate reader (Bio-Rad).

### 2.18 Transwell cell migration assays

PBMC from FAP subjects or healthy donors were activated with TransAct™ (1:100; Miltenyi-Biotec) and recombinant human IL-2 (100 IU/ml; PeproTech) in RPMI-1640 medium supplemented with GlutaMAX-I (Gibco), 5% human serum, 1 mM sodium pyruvate, nonessential amino acids, 10 mM HEPES, 1% penicillin-streptomycin (v/v). At day 5 post stimulation, 3x10^5^ T cells were deposited in the upper inserts of transwell plates (3 µm Pore size, Costar 3415) in RPMI-1640, 0.5% BSA (Nunc). The lower chambers contained the same medium supplemented or not with recombinant CXCL12 (R&D 350-NS; 5 ng/ml). After, 90 min of incubation at 37°C in 5% CO2, inserts were removed, and the same volume of input and migrated cell populations was analyzed by FACS. The percentage of CD4 and CD8 T cells was assessed.

### 2.19 Immunological synapse

Experiments were performed as previous described (16).

#### 2.19.1 Flat pseudo-synapses on anti-CD3-coated coverslips

Coverslips were washed with 70% HCl-ethanol and coated with Poly-L-lysine 0.002% in water (Sigma-Aldrich). Coverslips were further coated with anti-CD3 (10 μg/mL; UCHT1, BioLegend) overnight at 4°C, washed and blocked for 30 min at 37°C with human CD8 medium. T cells were plated on anti-CD3-coated coverslips for 5 min at 37°C and fixed with 4% paraformaldehyde for 13 min at room temperature. Microtubule detection required further 20 min incubation at -20°C in ice cold methanol. Cells on coverslips were then stained for1h at room temperature with anti-β-tubulin (5 μg/mL; Merck Millipore) Abs in PBS, 1% BSA, 0.05% Saponin. Coverslips were washed with PBS, 1% BSA, then incubated 45 min at room temperature with PBS, 1% BSA, 0.05% Saponin and fluorescent-coupled secondary Ab and Texas Red-X phalloidin (1/100; Invitrogen). After three washes in PBS with 1% BSA, coverslips were mounted on microscope slides using ProLong Gold Antifade mounting medium (Life Technologies).

#### 2.19.2 CTL-target cell immunological synapse

P815 tumor cells, previously coated with anti-CD3 (20 μg/ml; OKT3 produced by A. Alcover) for 45 min at 4°C, were plated on poly-L-lysine-coated coverslips for 15 min at 37°C. Coverslips were gently washed with PBS, and human CTLs were added at a 1:1 CTL-target ratio and incubated for 15 min at 37°C. Cells were then fixed with 4% paraformaldehyde for 13 min at room temperature. Coverslips were washed in PBS and incubated 1h in PBS with 1% bovine serum albumin (BSA) (v/v) to prevent unspecific binding. Cells were then incubated 1 h at room temperature with PBS, 1% BSA, 0.1% Triton X-100 and anti-centrin (1/100; Abcam), anti CD8 (UCHT-4, Sigma) Abs and Texas Red-X phalloidin (1/100; Invitrogen). Coverslips were then washed with PBS, 1% BSA and incubated 45 min at room temperature with the corresponding fluorescent-coupled secondary Ab and Texas Red-X phalloidin (1/100; Invitrogen). After three washes in PBS with 1% BSA, coverslips were mounted on microscope slides using ProLong Gold Antifade mounting medium (Life Technologies).

### 2.20 Confocal microscopy, image treatment and quantitative analysis

Confocal images were acquired with an LSM 700 confocal microscope (Zeiss) using the Plan-Apochromat 63x/1.40 NA objective. Optical confocal sections were acquired with ZEN software (Zeiss) by intercalating green and red laser excitation to minimize channel cross talk. All analyses were performed using Fiji software (22). For F-actin analysis, optical sections were acquired at 1 μm intervals. Formation of the actin ring was performed on one confocal section at the cell-coverslip contact. F-actin intensity profiles were assessed along a line drawn across each cell image. Ranking of each cell in a category (presence of F-actin ring and central clearance; low F-actin ring and low clearance; intermediate) was decided after observing actin profiles on 3-4 different angles. For microtubule pattern and synapses analyses, optical sections were acquired at 0.2 μm intervals and images were treated by deconvolution with the Huygens Pro Software (Scientific Volume Imaging). Microtubule pattern analysis was performed on projection of 4 confocal sections at the cell-coverslip contact as described below. T cell morphology in synapses was analyzed as described below. Centrosome localization was estimated by measuring the distance between the anti-pericentrin stained puncta and the CTL plasma membrane at the center area of the immunological synapse.

#### 2.20.1 Quantifying microtubule organization on flat immunological synapses

To quantify differential microtubule patterns in FAP *versus* healthy subject T cells, we developed a computer-based method implemented in Python to quantify microtubule radiality on T cells spread on anti-CD3-coated coverslips. The method comprises the following steps, summarized in **Supplementary Figures 4**, **1**) Segment cells with Otsu’s method (23) and remove small connected components. 2) Generate a radial and circular unit vector field from the center of mass computed from the convex hull of the segmentation mask obtained in the previous step. 3) Extract microtubule directionality vectors by estimating the dominant local orientation using a structure tensor (24). 4) Fit an elliptical Fourier descriptor (25) to the contour of the convex hull of the segmentation mask. 5) Compute intermediate elliptical Fourier descriptors between the outer edge of the convex hull and its center of mass. 6) Obtain radial sections in between these intermediate contours. 7) Compute the sum of absolute dot products between the microtubule vector field and both the radial and circular vector fields. 8) Estimate the *degree of radiality* (DoR) by dividing the former result by the latter. The DoR was plotted versus the relative distance to the cell center of mass. Code available at https://github.com/krentzd/fap_apc_image_analysis.

#### 2.20.2 Quantifying T cell morphology in immunological synapses of with anti-CD3-coated tumor target cells

All analyses were conducted on maximum intensity projections of image confocal stacks. Confocal microcopy images of T cell–target cell conjugates stained for CD8 (green) and F-actin (red) for three example cells (A-C) are shown in **Supplementary Figure 5**. Corresponding T cell masks generated by thresholding the CD8 signal are displayed in white overlapped to their convex hull in grey and the fitted major axis (MA) in blue (D-F). The roughness of the cell was measured as the ratio of the perimeter of the cell to the perimeter of its convex hull (26). Higher values indicate higher degrees of cell surface roughness. The roundness was calculated as (4 x T cell mask area)/(π x MA^2^). A roundness closer to 1.0 indicates a shape closer to a perfect circle. While the metrics are not completely unrelated, roundness is less sensitive to local irregularities being based on the area (27) and thus can be seen as generally providing information on the cell larger scale deviation from a round phenotype. Roughness, on the other hand, better captures variations in small scale irregularities of the cell surface. All analyses were conducted using Python 3.10.8 using the regionprops method from scikit-image version 1.23.5. The Crofton perimeter was used, which approximates the perimeter based on the Crofton method in four directions (28). The MA is defined as the length of the major axis of an ellipse with the same normalized second central moment as the mask.

### 2.21 Statistics

Statistical analyses were carried out as follows: Whole blood immunophenotyping data were analyzed using the Wilcoxon rank test using Prism software (GraphPad). In graphs, each point represents one subject. Pairs of FAP and healthy subjects matched in sex and age are linked by a line. Whole blood gene expression by Nanostring and secretome by Luminex were analyzed by the T-test using Qlucore software. Due to the small sample size of this rare disease cohort, we approached our analysis in a discovery-based way and did not apply FDR correction to our p values for this analysis. Migration assays were analyzed by paired T-test, using Prism software (GraphPad). Cell proliferation, differentiation and cytokine production data on purified T cells were analyzed by the Wilcoxon rank test using Prism software (GraphPad). Killing and degranulation, and actin cytoskeleton reorganization were analyzed by two-way ANOVA test, using Prism software (GraphPad). Microtubule pattern and synapse morphology were analyzed by T-test for independent samples using Python software (version 3.10.8), SciPy (version 1.10.0) package (ttest_ind method https://docs.scipy.org/doc/scipy/reference/generated/scipy.stats.ttest_ind.html).

## 3 Results

To investigate the capacity of FAP subjects to mount immune responses we used an array of experimental approaches following the workflow displayed in **Figure 1**. First, we biochemically characterized the APC protein in FAP subjects using EBV-transformed cell lines derived from peripheral blood mononuclear cells. Second, analyses on whole blood samples provided us with a wide view on immune cell populations, gene expression and chemokine/cytokine production in response to diverse stimuli. Finally, we investigated the capacity of purified T cells to proliferate, differentiate, migrate and perform effector functions *in vitro*. APC involvement in these functions was inferred from our previous work on APC-silenced human T cells and/or *Apc^Min/+^* mutant mice (15–17).

### 3.1 APC gene mutations and APC protein expression in FAP subjects’ immune cells

**Figure 2:**
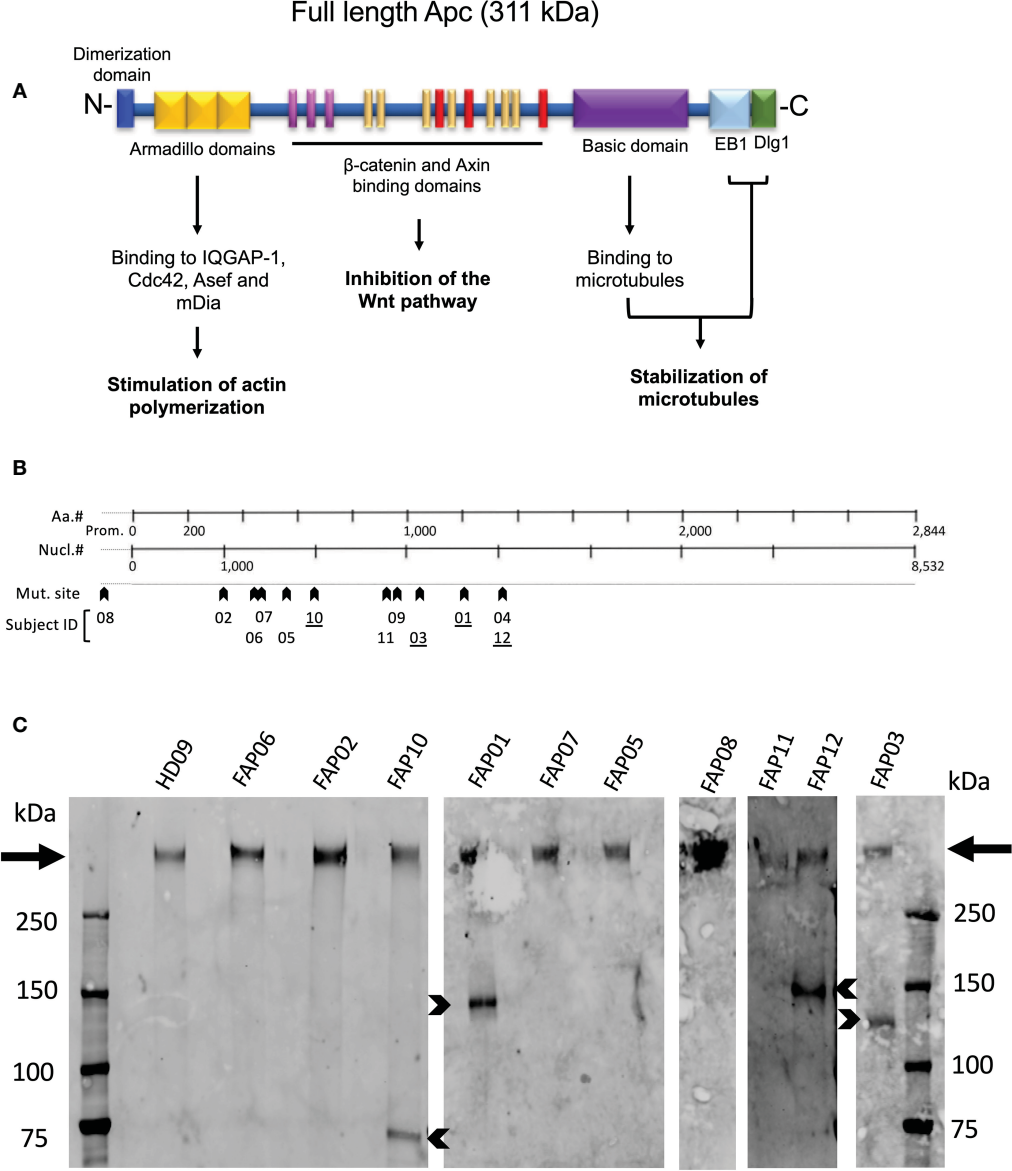
APC protein expression in FAP and healthy subjects. **(A)** Schematic representation of the functional domains of the APC protein. **(B)** Localization of APC gene mutations of FAP subjects investigated here. **(C)** Western blots of anti-APC immunoprecipitates obtained from EBV-transformed cells from, 10 FAP and one healthy subjects. Arrows point to the full-length band. Arrowheads point to truncated APC polypeptides expressed by some FAP subjects (underlined numbers in B). #, number.

**Figure 2A** schematically shows APC protein functional domains. Most mutations carried by FAP subjects investigated here were localized between nucleotide 1000 and 4000 (**Figure 2B**, arrowheads), a gene region in which many APC mutations in FAP subjects have been previously reported (29). Excepted one FAP subject who harbored a mutation in the promoter region, the other FAP subjects carried different mutations in the coding region. APC mutations included missense, nonsense with premature stop codon and frameshift mutations potentially leading to a lack of protein expression of the mutated allele or expression of truncated forms. However, the actual protein state is not fully predictable.

We therefore analyzed APC protein expression by biochemical approaches. Western blots of whole T cell lysates were not sensitive enough to detect APC. We therefore implemented the assay using EBV-transformed B cell lines generated from each subject that provided us with a larger number of cells to prepare lysates, and a VHH anti-APC Ab, which allowed us to efficiently enrich APC by immunoprecipitation prior to performing western blots. All samples from both FAP and healthy subjects displayed a main band of a molecular weight corresponding to the full-length protein (311 kDa) (**Figure 2C**, arrows). This was expected as FAP subjects harbored heterozygous mutations. Interestingly, four FAP subjects displayed truncated forms of different apparent molecular mass (**Figure 2C**, arrowheads). APC protein expression appeared variable, and we could not discriminate differences between the healthy and FAP subjects full length protein forms.

### 3.2 Whole blood immunophenotyping unveils differences in immune cell populations in FAP *versus* healthy subjects

**Figure 3:**
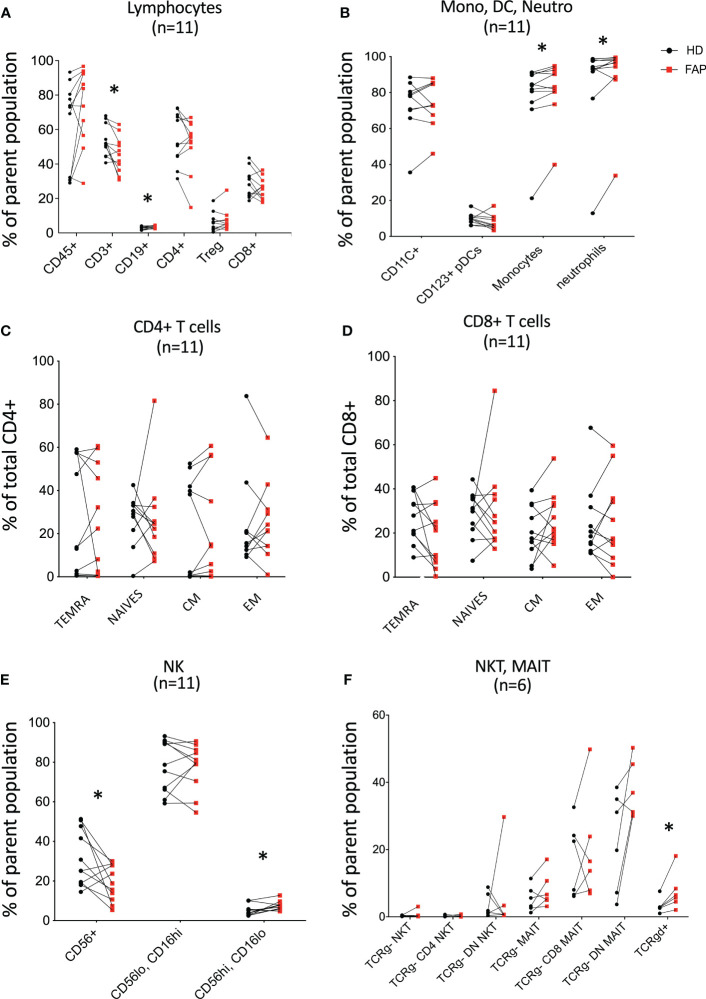
Whole blood immunophenotyping by multiparameter flow cytometry. Whole blood was prepared, and immune cells were stained with directly coupled fluorescent antibodies as detailed in materials and methods and in **Supplementary Table 1**. Cells were analyzed by multiparameter flow cytometry, following the gating strategies described in **Supplementary Figures 1**, **2**. Cell surface molecules defining cell populations are depicted in **Supplementary Figures 1**, **2** that include: **(A)** lymphocytes. **(B)** monocytes (Mono), dendritic cells (DC), neutrophils (Neutro). **(C, D)** CD4 and CD8 T cell subsets. **(E, F)** NK, NKT, MAIT, Tγδ cells. Each pair of sex and age matched FAP and healthy subject is linked by a line. Each dot represents a subject. Red square and black round symbols correspond to FAP and healthy subjects, respectively. Percentage of cells expressing the characteristic markers is plotted. Significance was determined by the Wilcoxon rank test. N = number of pairs. The *p* values are represented as follows: *p < 0.05, non-significant, *p* ≥ 0.05.

To obtain a general view of the cellular immune system in FAP subjects *versus* age and sex-matched healthy donors, we analyzed the expression of an array of cell surface proteins defining the various immune cell lineages and populations by multi parameter flow cytometry. Gating strategies are shown in **Supplementary Figures 1-3**. T cell and NK cell populations were studied in more detail to distinguish different subpopulations. Results for each pair of FAP and matched healthy subjects are shown in **Figure 3**; **Supplementary Figure 6**.

Most immune cell populations showed no differences between FAP and healthy subjects, although some significant differences were observed and summarized in **Table 1**. These included the percentages of total T cells (CD3+), and CD56+ NK cells which were diminished, whereas B cells (CD19+), CD16+ monocytes, neutrophils (CD16+CD66b+) and γδ T cells were slightly increased in FAP patients (**Figures 3A, B, E, F** **)**. Other interesting trends were observed, although they were not statistically significant, e.g. the percentage of MAIT cells (CD3+, TCRγδ-, CD4/CD8 DN, TCRα7.2+, CD161+) was increased in most of the FAP subjects with respect to their matched healthy subject (**Figure 3F**). The results on NK cells, prompted us to further explore NK populations by analyzing expression of NK surface molecules. We observed some significant differences between FAP and healthy subjects within some NK cell populations (**Supplementary Figures 7-9**, summarized in **Table 1**). The differences in the number of subjects between graphs is due to technical problems that prevented the exploitation of some experiments.

**Table 1:** Immune cell populations displaying significant changes between FAP and healthy subjects.

Cell population markers	Change	Figure
T cells, CD3+	Down*	**3A**
B cells, CD19+	Up*	**3A**
T cells, TCRgd+	Up*	**3F**
Monocytes CD16+ CD14+	Up*	**3B**
Neutrophils, CD16+ CD66b+	Up*	**3B**
NK cells, CD56+	Down*	**3E**
NK CD56hi CD16lo	Up*	**3E**
NK, CD226- NKp44+	Down*	**Sup Fig 7F**
NK, CD226- NKp44-	Up*	**Sup Fig 7F**
NK, CD158el- CD158el/e2-	Down**	**Sup Fig 8C**
NK, CD158el+ CD160+	Up**	**Sup Fig 8D**
NK, CD158el+ CD160-	Up*	**Sup Fig 8D**
NK, CD158el+ NK.p46-	Up**	**Sup Fig 8D**
NK, CD158el/e2+ NKG2a-	Up*	**Sup Fig 8E**
NK, CD158el/e2+ CD160-	Up*	**Sup Fig 8E**
NK, 2B4+ CD158a-	Down*	**Sup Fig 9F**

Summary extracted from whole blood immunophenotyping figures depicted in column 3. Wilcoxon rank test: **<0.01, * p<0.05. Up/Down = Upregulated/Downregulated expression in FAP subject cells with respect to healthy subject cells.

Therefore, these results indicate that FAP subjects may have mild but significant differences in T and NK cell populations.

### 3.3 Differential immune gene expression profiles in FAP subjects in *ex vivo*-stimulated whole blood

**Figure 4:**
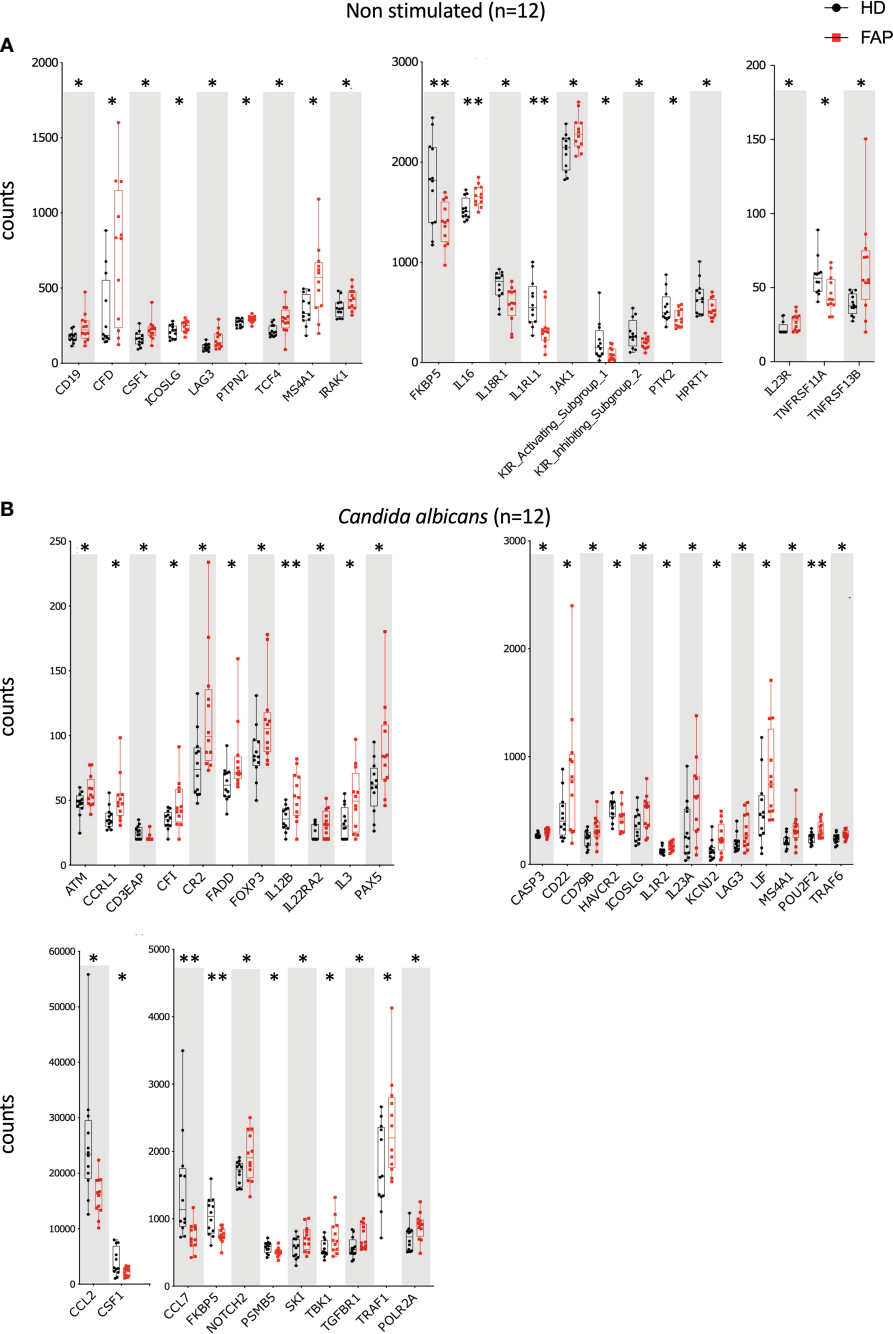
Whole blood immune cell gene expression. Whole blood from pairs of sex and age matched FAP and healthy subjects (one pair per experiment) was added to TruCulture tubes with no stimulus, *Candida albicans*, SEB, or anti-CD3+anti-CD28 stimuli and incubated during 22 hours at 37°C. Cells and supernatants were then separated. Cells were lysed and mRNA was frozen. The 12 pairs of FAP and healthy subjects were then analyzed together by NanoString technology using a panel of 579 immune related genes listed in **Supplementary Table 2**. Cells were non-stimulated **(A)** or received *Candida albicans* stimulation **(B)**. Data were analyzed using Qlucore software. Only pairs displaying significant differences (p<0.05) are represented. Black round dots correspond to healthy subjects and red square dots correspond to FAP subjects. Whiskers represent minimum and maximum values, whereas boxes represent second and third quartiles framing the median. Each dot represents one subject. n = number of pairs. The *p* values (t-test) are represented as follows: **p < 0.01, *p < 0.05.

**Figure 5:**
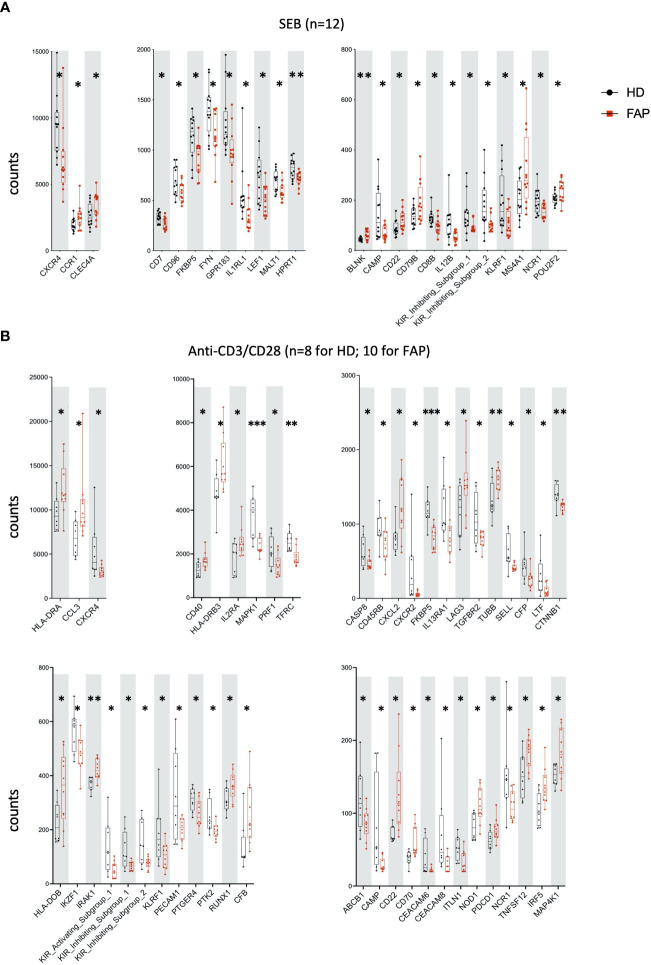
Whole blood immune cell gene expression. Samples were processed as for **Figure 4**. **(A)** SEB stimulation. **(B)** Anti-CD3+anti-CD28 stimulation. Data were analyzed using Qlucore software. Only pairs displaying significant differences (p<0.05) are represented. Black round dots correspond to healthy subjects and red square dots correspond to FAP subjects. Whiskers represent minimum and maximum values, whereas boxes represent second and third quartiles framing the median. Each dot represents one subject. n = number of pairs. The *p* values (t-test) are represented as follows: ***p < 0.001, **p < 0.01, *p < 0.05. Data of non-responders to anti-CD3 + anti-CD28 were not included in the analysis.

Whole blood from FAP subjects and their matched healthy controls was stimulated in TruCulture^®^ tubes containing no stimuli (ns), *Candida albicans*, *Staphylococcus enterotoxin* B (SEB) superantigen and anti-CD3+anti-CD28 Abs, as described in methods. The expression of 579 genes of the immune response panel (**Supplementary Table 2**) were analyzed by the NanoString technology. Samples from 12 pairs of FAP and matched healthy control subjects were analyzed. One hundred genes were found differentially expressed, upregulated or downregulated, in FAP *versus* healthy subjects in non-stimulated samples (20 genes) or under one of the three stimulation conditions, *C. albicans* (35 genes), SEB (24 genes) and CD3/CD28 (47 genes) (p<0.05) (**Figures 4**, **5**; **Supplementary Table 4**). Most of the genes that were differentially expressed across the stimuli were upregulated under non-stimulated or *C. albicans* stimulation but downregulated under SEB or CD3/CD28 stimulation. The overlap between genes affected by several stimulation conditions was low. Only one gene, FKBP5 was downregulated under the four conditions, and only 6 genes were downregulated by both T cell stimuli, SEB and CD3/CD28 (i.e. CXCR4, KIR_IS_1/2, KLRF1, CAMP, NCR1). Other genes could also be individually pinpointed, including B and T cell proteins, as we comment in the discussion section. False discovery rate correction gave q values higher than 0.05. Therefore, we consider these results as an initial discovery phase due to the small sample size of this rare disease cohort, which will require future replication in other patient cohorts, but may orient future investigations.

### 3.4 Enhanced secreted protein profile in FAP subjects in response to T cell stimulation

**Figure 6:**
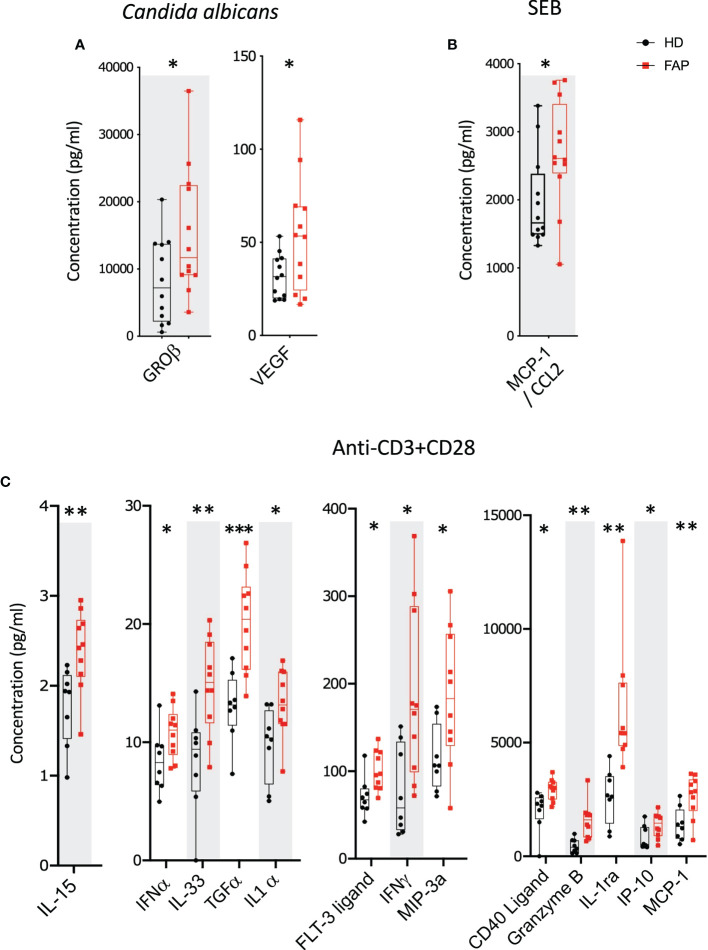
Whole blood secreted proteins after stimulation. Whole blood from pairs of sex and age matched FAP and healthy subjects (one pair per incubation) was added to TruCulture tubes with no stimulus, *Candida albicans*, SEB, or anti-CD3+anti-CD28 stimuli and incubated during 22 hours at 37°C. Cells and supernatants were then separated. Supernatants were kept frozen. All 12 pairs of FAP and healthy subjects were then analyzed together by Luminex technology using a panel of 44 analytes (**Supplementary Table 3**). **(A)** *Candida albicans* stimulation. **(B)** SEB stimulation. **(C)** Anti-CD3+anti-CD28 stimulation. Data were analyzed using Qlucore software. Only pairs displaying significant differences (p<0.05) are represented. No significant differences were observed in non-stimulated cells that secreted very low levels of these proteins. Black round dots correspond to healthy subjects and red square dots correspond to FAP subjects. Whiskers represent minimum and maximum values, whereas boxes represent second and third quartiles framing the median. Each dot represents one subject. n = number of pairs. The *p* values (t-test) are represented as follows: ***p < 0.001, **p < 0.01, *p < 0.05. Data from non-responders to anti-CD3+anti-CD28 were not included in the analysis.

Whole blood from FAP patients and their matched healthy controls were stimulated in TruCulture^®^ tubes containing no stimuli, SEB, anti-CD3+anti-CD28 Abs or *C. albicans*, as described in methods. The production of 44 immune response-related secreted proteins was analyzed by Luminex technology. The strongest effect was observed with anti-CD3+CD28-stimulated cells in which 13 proteins were expressed at differential levels in FAP *versus* healthy subjects. Only two and one secreted proteins were found differentially produced under *C. albicans* and SEB stimulation, respectively. Interestingly, under all three stimulation conditions, proteins differentially secreted by cells from FAP subjects were upregulated with respect to healthy subject cells (**Figure 6**; **Supplementary Figure 10, 11**). False discovery rate correction gave q values higher than 0.05. Therefore, as for the transcriptome analysis, we consider these results as an initial discovery phase due to the small sample size of this rare disease cohort, which will require future investigation in other patient cohorts.

**Figure 7:**
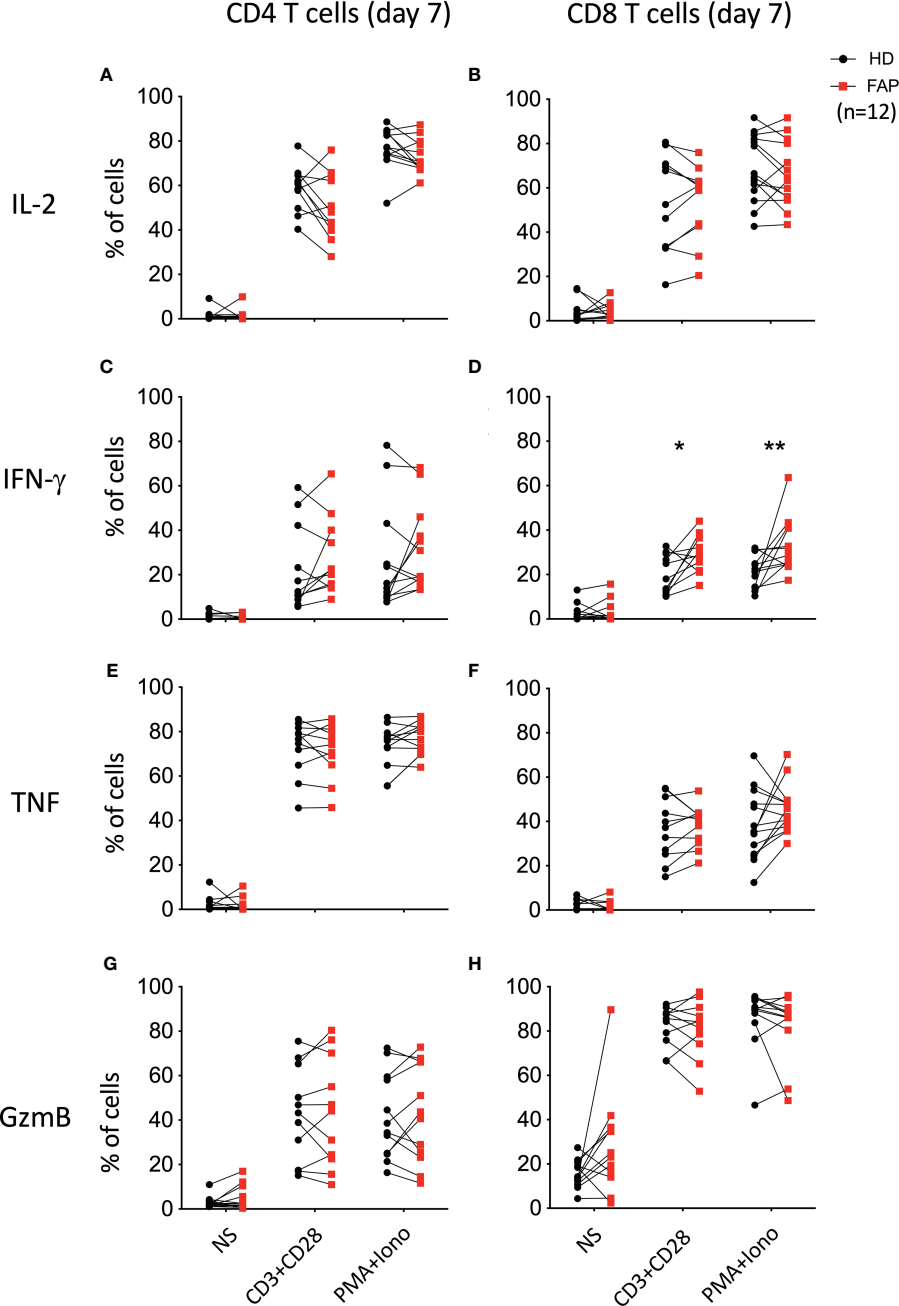
Cytokine production by *in vitro* expanded peripheral blood T cells. T cells were expanded *in vitro* for 7 days by activation with anti-CD3+CD28 and cultured with IL-2. At day 7, cells were washed and restimulated for 6 h with anti-CD3+CD28 or with PMA+ionomycin. CD4^+^ and CD8^+^ T cells expressing IL-2, IFN-γ and TNF were monitored by intracellular cytokine staining and flow cytometry. In parallel, granzyme B expression was monitored. Plots show the percentage of CD4^+^ and CD8^+^ T cells expressing IL-2 **(A, B)**, IFN-γ **(C, D)**, TNF **(E, F)** and granzyme-B (GzmB; **G, H**). Pairs of sex and age matched FAP and healthy subjects are shown linked by a line. Each dot represents a subject. Round black dots correspond to healthy subject and red, square dots correspond to FAP subjects. n = number of pairs. Significance was determined by the Wilcoxon rank test. The *p* values are represented as follows: **p < 0.01, *p < 0.05.

To further explore whether the differences in cytokine production observed under CD3-CD28 stimulation were due to a specific effect on T cell populations, freshly isolated PBMCs were activated for 6 h with anti-CD3+anti-CD28 Abs, or with PMA/ionomycin. In addition, T cells were expanded *in vitro* for 7 days upon activation with anti-CD3+anti-CD28 and cultured in IL2. At day 7, cells were washed to remove IL-2 and restimulated for 6 h with anti-CD3+anti-CD28 or with PMA+ionomycin. CD4+ and CD8+ T cells expressing IL-2, IFN-γ and TNF were monitored by intracellular cytokine staining and flow cytometry. In parallel, we monitored granzyme B expression to assess the capacity of T cells to become cytotoxic (**Figure 7**). Stimulation at day 0 did not reveal significant differences between T cells from FAP and healthy subjects (**Supplementary Figure 12**). However, when T cells were restimulated after 7 days in culture, we observed a higher percentage of CD8+ T cells from FAP subjects producing IFN-γ (**Figure 7D**; **Supplementary Figure 13**).

Therefore, whole blood stimulation in TruCulture tubes appears as a more sensitive approach to reveal differences between FAP and healthy subjects, suggesting that complex interactions between immune cells are involved.

### 3.5 *In vitro* T cell growth and differentiation is similar in FAP and healthy subjects

**Figure 8:**
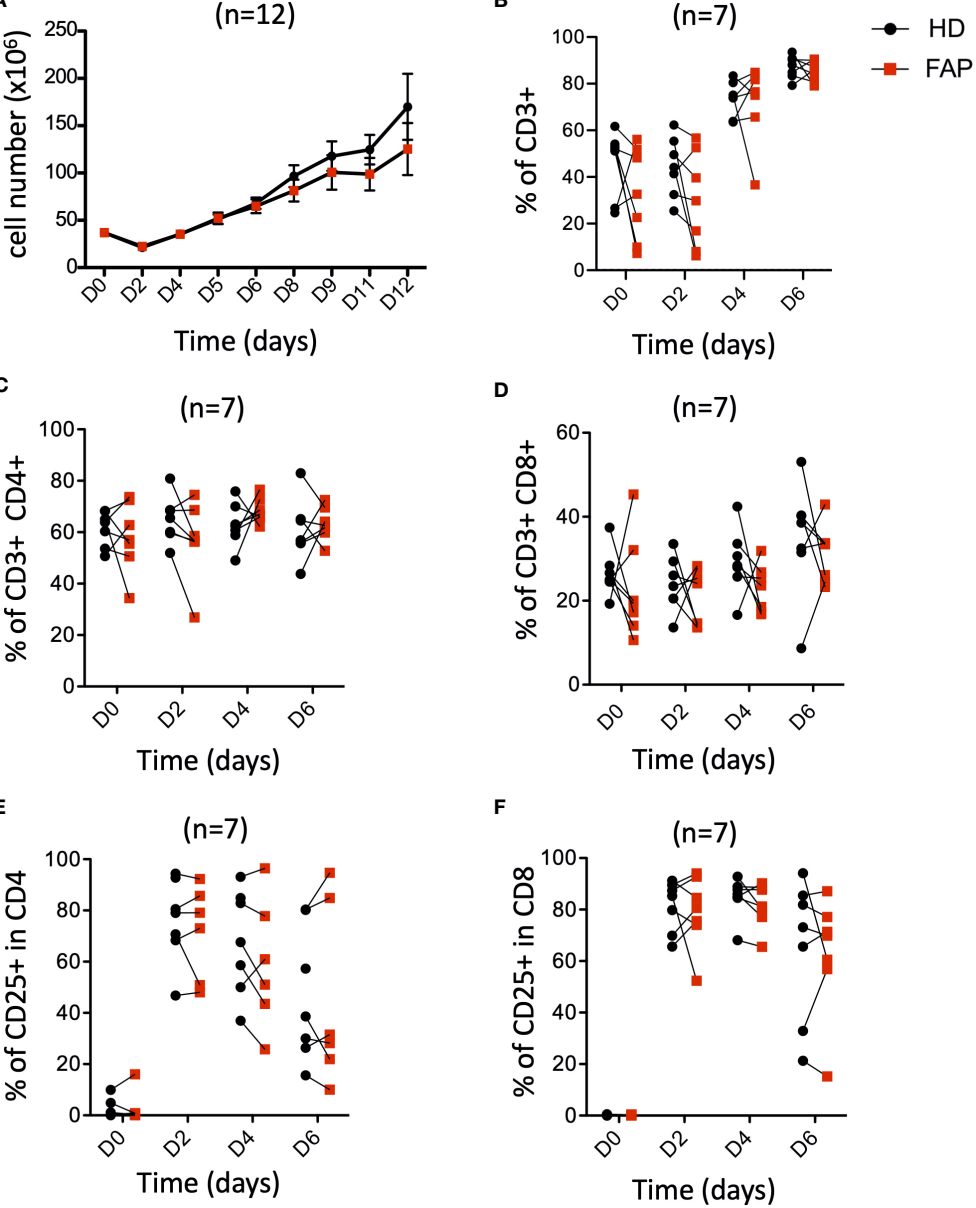
Peripheral blood T cell proliferation and activation. PBMCs from FAP or healthy subjects were activated with anti-CD3+CD28 Abs and IL-2 and their proliferation and expression of the activation marker CD25 were monitored by flow cytometry at various times (Day 0-6). Pairs of sex and age matched FP and healthy subjects are shown linked by a line. **(A)** cell counts. **(B)** Percentage of CD3^+^ T cells. **(C, D)** Percentage of CD4+ and CD8+ T cells. **(E, F)** Percentage of CD25^+^ cells among CD4^+^ and CD8^+^ T cells. Each dot represents a subject. Round black dots correspond to healthy subject and red, square dots correspond to FAP subjects. n = number of pairs. Significance was determined by the Wilcoxon rank test. No significant differences were found, p > 0.05.

*APC* mutations lead to defects in growth and differentiation of intestinal epithelial cells, leading to polyposis. This is due to the implication of APC in the control of the Wnt/β-catenin signaling pathway (30, 31). This prompted us to investigate whether T cell growth and differentiation could be altered in FAP subjects. PBMCs were stimulated *in vitro* with anti-CD3+CD28 antibodies and IL-2 and their proliferation and expression of differentiation markers were monitored at various times. The increase in cell number (**Figure 8A**), as well as the percentage of total CD3+, CD4+ and CD8+ T cells were similar between FAP and healthy subjects. Moreover, activation, as assessed by the cell surface expression of CD25 was comparable (**Figures 8B-F**).

Finally, we investigated the capacity of T cells to differentiate from naïve to central- and effector-memory populations and to CTL, following *in vitro* activation. To this end, we analyzed the differential cell surface expression of CCR7 and CD45RA markers and the expression of granzyme B. Responses were quite variable in the two groups of subjects and no significant differences could be demonstrated between FAP and healthy subject cells (**Supplementary Figure 14**), suggesting that main pathways of T cell growth and differentiation were not strongly affected *in vitro*.

### 3.6 Cytotoxic activity by *in vitro*-differentiated CTLs is similar in FAP and healthy subjects

**Figure 9:**
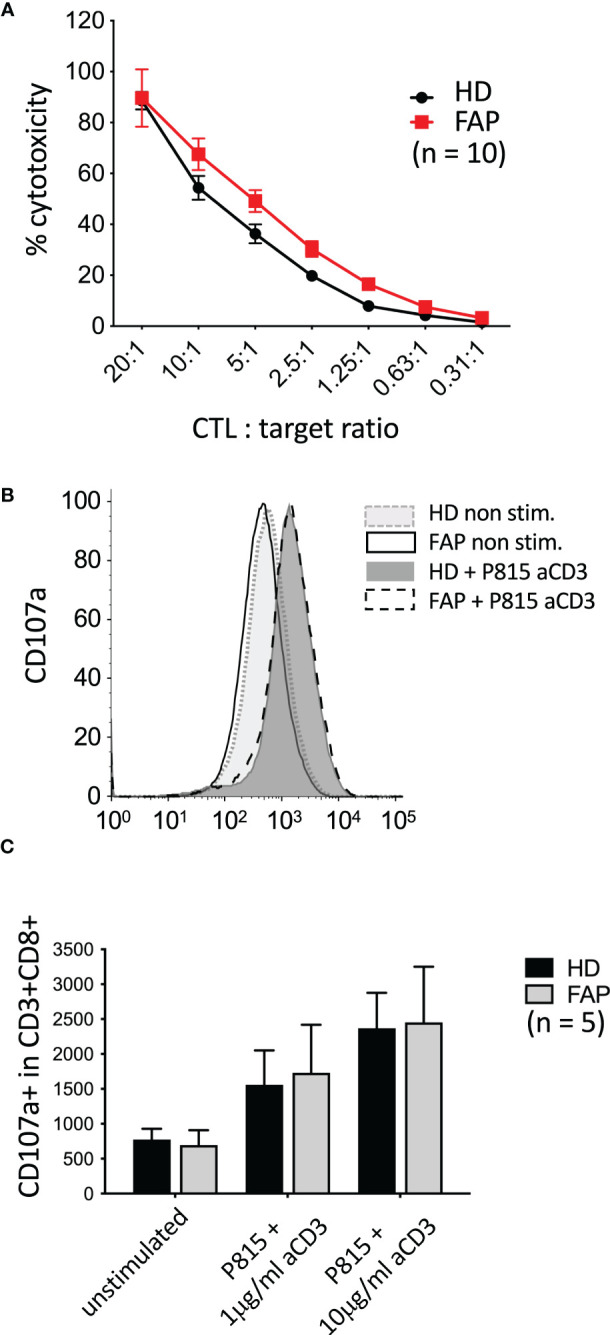
Killing activity and degranulation by *in vitro*-differentiated CTL *In vitro*-differentiated CTLs were assayed for their capacity to: **(A)** kill anti-CD3-coated P815 tumor cells during 3 h, as assessed by a colorimetric assay. **(B, C)** degranulate, as assessed by anti-LAMP1 (CD107a) antibody staining by flow cytometry. **(B)** Example of FACs plots from a single pair of sex and age matched FAP and healthy subjects. No significant differences between FAP and healthy subjects were observed, either in cytotoxicity or in degranulation. Significance was determined by the 2-way ANOVA (**A**, mean ± SD, n=10) or multiple Mann-Whitney (**C**, mean ± SD, n=5) tests. No significant differences were found, *p* ≥ 0.05.

We have previously shown that APC silencing in human T cells or APC mutation in *Apc^Min/+^* mice mildly impaired CTL cytotoxicity as assessed *in vitro* assays, although degranulation seemed not to be affected (16). Therefore, we investigated whether these CTL functions could be altered in FAP subjects. To this end, *in vitro*-differentiated CTLs were assayed for the capacity to kill anti-CD3-coated P815 tumor cells, and to degranulate, as assessed by anti-LAMP1 (CD107a) antibody staining. No significant differences between FAP and healthy subjects were observed (**Figure 9**).

### 3.7 FAP subjects CTLs form less structured immunological synapses

**Figure 10:**
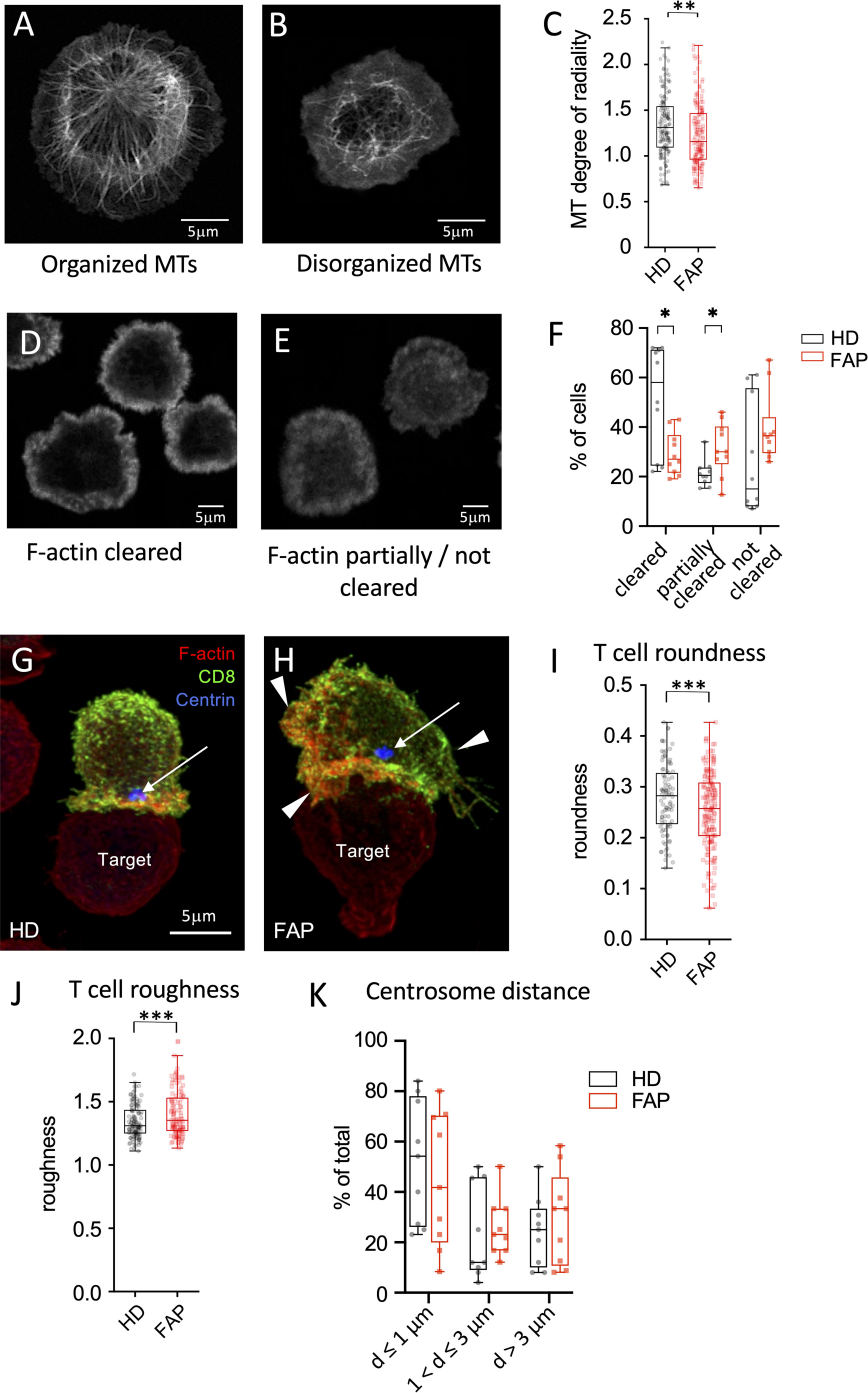
Immunological synapse organization by *in vitro*-differentiated CTL **(A-F)** *In vitro* differentiated CTLs were stimulated on anti-CD3-coated coverslips for 5 min at 37°C to generate flat immunological pseudo-synapses. Cells were then fixed and stained with anti-β-tubulin Abs to reveal microtubules **(A, B)** or fluorescent-coupled phalloidin to reveal filamentous (F)-actin **(D,E)**, as described in Methods. **(A, B)** Confocal optical sections were acquired at 0.2 µm intervals. Images were post-treated by deconvolution. A 0.8 µm section at the cell–coverslip contact is shown. **(C, D)** Confocal optical sections were acquired at 1µm intervals. A single section at the cell–coverslip contact is shown. **(A-C)** Microtubule network organization. **(A)** an example of cell with a radial plane of microtubules close to the contact site with visible microtubule organizing center, mainly found in healthy subject cells. **(B)** an example of cell with non-radial, rather disorganized microtubule pattern lacking microtubule organizing center, more often observed in FAP subjects. **(C)** Quantitative image analysis of microtubule radial patterns was performed as described in methods. Box and whiskers plot represents the degree of radiality of microtubules calculated at relative radial distance of 2 (see **Supplementary Figure 4**). The box extends from the first to the third quartile of the data, with a line at the median. The whiskers extend from the box by 1.5x the inter-quartile range. n=162 HD or 160 FAP cells, respectively, from 7 subjects per group. Significance was determined by t-test, ** *p* < 0.01. Scale bar, 5 µm. **(D-F)** F-actin organization patterns: **(D)** pattern corresponding to centrally depleted F-actin and accumulation at the periphery of the synapse (F-actin cleared). **(E)** pattern corresponding to lower depletion of F-actin at the center of the synapse with more often presence of F-actin in the synapse center (F-actin partially or not cleared). **(F)** Image analysis was performed by blind pattern ranking by three independent investigators. Box and whisker plot represent mean ranking from 10 independent experiments, each including a pair FAP and a healthy subject. The box extends from the first to the third quartile of the data, with a line at the median. The whiskers extend from minimum to maximum values. Significance was determined by Mann-Whitney test, *p < 0.05. **(G-K)** *Ex vivo*-differentiated CTLs from healthy or FAP subjects were incubated with anti-CD3–coated P815 target cells, for 15 min. Cells were then fixed and stained with anti-CD8 Ab (green), phalloidin (red) to label F-actin, anti-pericentrin Ab to label the centrosome (blue, arrow). Confocal optical sections were acquired at 0.2 µm intervals. Images were post-treated by deconvolution. Conjugate 3D reconstructions are shown. **(G)** Example of immunological synapse displaying a symmetric configuration according to a vertical axis (vertical line), and with polarized centrosome apposed to the synapse (blue spot pointed by an arrow), more often found in healthy subject cells. Scale bar, 5 µm. **(H)** Example of immunological synapse showing asymmetric configuration and large membrane protrusions pointed by arrowheads. **(I, J)** Morphological changes were assessed by computer-assisted measurements of roundness and roughness as described in Methods. Box and whisker plot is as described in C, n=125 cells for healthy and 116 cells for FAP group, each composed of 6 FAP and healthy subjects. Significance was determined by t-test, ***p < 0.001. Scale bar, 5 µm. **(K)** Centrosome distance from the center of the synapse was measured using Fiji software. Plots represent percentage of cells displaying centrosome distances lower or equal to 1µm, between 1 and 3 µm and longer than 3µm. Box and whisker plot represent mean distance from 9 independent experiments, each including a pair FAP and a healthy subject. The box extends from the first to the third quartile of the data, with a line at the median. The whiskers extend from minimum to maximum values. Significance was determined by Mann-Whitney test.

We previously showed that APC silencing impairs T cell polarization and immunological synapse formation of CD4 and CD8 T cells (15, 16). Several features were observed using two different experimental setups. In the first one, T cells were activated on anti-CD3-coated coverslips forming flat pseudo-immunological synapses in which reorganization of the microtubule and actin cytoskeletons was monitored. Microtubule cytoskeleton polarizes at the pseudo-synapse, forming a finely organized radial network extending from the centrosome towards the synapse edges (15, 16) (**Figure 10A**). Moreover, actin actively polymerizes at the pseudo-synapse and filamentous (F)-actin initially covers most of the T cell contact site, then forming a peripheral ring (**Figure 10D**) that eventually closes back (32, 33). Cytotoxic granules, transported by microtubules, are secreted inside the F-actin ring, where F-actin is less present (32, 33). We previously showed that both microtubules and F-actin patterns were affected in APC-silenced T cells. Centrosome polarization and radial microtubule patterns were altered, similarly to **Figure 10B**, while F-actin clearance from the synapse center was less efficient, similarly to **Figure 10E**. As a possible consequence, polarized lytic granule secretion at the synapse was impaired (16).

In line with these findings, we found that microtubule network organization at the synapse of T cell blasts, as assessed by the radial organization of microtubules along the synapse (**Supplementary Figures 4**), was altered in T cells from FAP patients (**Figure 10C**). Variability among individual cells was higher and the radial organization less evident in FAP *versus* healthy subjects. Moreover, F-actin clearance from the center of the synapse was less efficient in T cells from FAP *versus* healthy subjects (**Figures 10D-F**).

In the second experimental setup, peripheral blood T cells differentiated *in vitro* into CTLs were put in contact with anti-CD3-coated P815 tumor cells and synapse formation observed. Our previous data showed that APC-silenced cells formed less polarized and less symmetric synapses, with more irregular T cell shapes and less efficient centrosome relocalization to the synapse (16). Here, we observed that the morphology of CTLs from FAP subjects, assessed by computer-based measurement of roundness and roughness (**Supplementary Figure 5**) displayed more irregular shapes (**Figures 10G-J**). Centrosome polarization, assessed by the distance from the center of the synapse was highly variable and not significantly different between T cells from FAP and healthy subjects (**Figure 10K**).

Hence, in line with previous data on APC-silenced cells, T cells from FAP subjects tend to form less structured immunological synapses. However, the high variability among individuals and the low number of subjects makes these findings indicative at this point.

### 3.8 T cell migration in response to chemokines is impaired in FAP subjects

**Figure 11:**
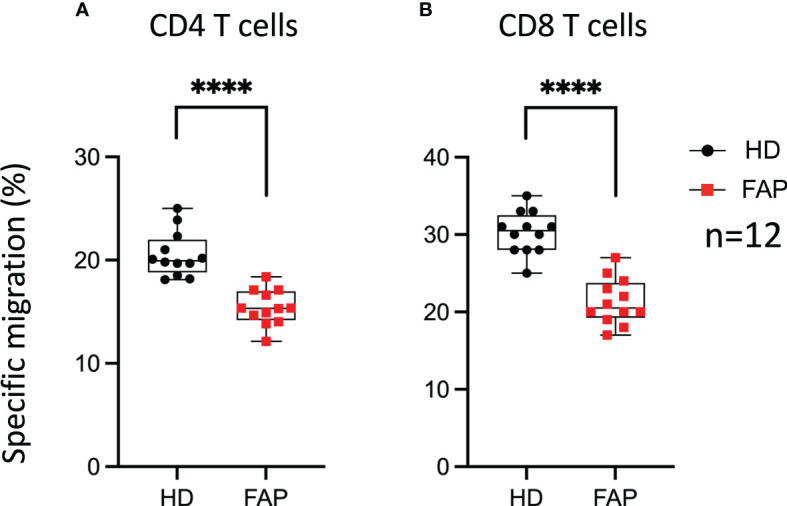
T cell migration in response to chemokine. PBMC from FAP or healthy subjects were activated with TransAct™ and IL-2. Five days post stimulation, T cells were placed in the upper chamber of transwell migration filters (3 µm pore size) and let to migrate towards CXCL12 (5 ng/ml) containing medium in the lower chamber for 90 min. The percentage of input and migrated CD4^+^ and CD8^+^ T cells was assessed by FACS. **(A, B)** Round black dots correspond to healthy subject and red, square dots correspond to FAP subjects. Whiskers represent minimum and maximum values, whereas black and red boxes represent the second and third quartiles framing the median. Significance was determined by Mann-Whitney paired t-test. n = number of pairs. The *p* values: ****p < 0.0001.

We have previously shown that APC integrity is necessary for T cell migration in response to chemokines, as assessed in APC-silenced CEM tumor T cells and in T cells from a subset of the FAP subjects analyzed in this study (17). This is consistent with the role of APC as polarity regulator in a variety of cells including T lymphocytes (6, 34). Here, we extended our analyses to T cells from all 12 FAP subjects and their matched healthy controls and confirmed that T cells from FAP subjects were significantly less efficient to migrate through pores in response to CXCL12 chemokine (**Figure 11**).

Therefore, T cell migration was the most consistent feature differentiating healthy from FAP subject immune responses, under our experimental conditions.

## 4 Discussion

The aim of this study was to obtain insight into the potential impact of APC mutations on immune cell responses in FAP subjects, which could condition their antitumor defense. Strong changes in the presence of immune cell populations and/or in their functions were not expected, since no immunodeficiencies have been previously associated with FAP. However, considering our previous findings showing the implication of APC in T cell functions (15–17), it was important to investigate whether some discrete or combined mild immune cell alterations could suggest an effect on antitumor immune defense. To this end, we have examined an array of immune cell response features in peripheral blood cells. In each assay, we compared one FAP with one sex and age-matched healthy subject. Two types of experiments were performed (**Figure 1**), one set based on whole blood analyses that provided a nonbiased approach to investigate immune cell responses *ex vivo*. A second set of assays was performed on PBMC or purified T cells and focused on T cell features in which we had previously observed functional effects of APC silencing (15–17).

To compare APC protein expression to potential effects in immune cell responses, we carried out biochemical analysis of APC protein expressed by FAP subjects. This was challenging because of the low levels of APC protein expression in PBMC and the poor performance of available APC Abs. We therefore generated EBV transformed B cell lines allowing us to have larger amounts of cell protein extracts, and we developed a high affinity VHH Ab, allowing us to carry out immunoprecipitation to enrich APC protein from cell lysates. In this way, we could analyze APC protein expression in 10 out of 12 FAP subjects. All of them expressed a full-length protein, consistent with heterozygosity of APC mutation in blood lymphocytes. Moreover, four of them, expressed truncated forms of different molecular mass. Bands corresponding to the full-length protein species appear quite variable in intensity and no clear differences were observed between the healthy subject and the FAP subjects.

Immunophenotyping carried out by multiparameter spectral flow cytometry of whole blood showed a similar balance between most immune cell populations in FAP and healthy subjects with no major differences in the phenotype of peripheral blood immune cell populations. However, some differences could be observed in T cells (total CD3+) and NK cells (total CD56+), whose percentages were significatively reduced in FAP subjects. This could affect anti-tumor response in these individuals and prompted us to investigate in more detail the subpopulations of T and NK cells. We observed some significant differences among the percentages of several NK cell subpopulations, which were either decreased or increased with respect to healthy subjects (**Figure 3E**; **Supplementary Figures 7-9**, summarized in **Table 1**). However, no apparent differences were observed among the different T cell subpopulations. In contrast, the percentages of other cell populations were increased, including B cells, monocytes, neutrophils and γδ-T cells (**Figures 3A, B, F**; **Table 1**). We observed an increase of the CD14+CD16+ circulating monocytes. Two main populations of circulating monocytes have been described by the expression of CD14 and CD16 markers (CD14++ or CD14+/CD16+). Anti-tumor activities of monocytes and macrophages are well-known in particular the double positive population (35). However, CD14+CD16+ monocytes could also have pro-tumorigenic properties, likely due to their proangiogenic activity (36). Finally, increased levels of circulating CD14+CD16+ are observed in some epithelial cancers and were suggested as markers of aggressive tumors (37). Although understanding the potential clinical significance of the observed differences needs further investigation, our findings suggest that the balance between some immune cell populations is altered in FAP subjects and might affect the fine tuning of immune responses. However, we cannot rule out at this stage, that the differences observed might be a consequence of APC mutation, polyposis or of the surgical treatment undergone by these subjects, which may increase inflammation.

A transcriptomic analysis targeting 579 immune response genes in whole blood incubated with none, adaptive, or innate immunity stimuli in TruCulture tubes revealed about 100 differentially expressed genes between FAP and healthy subjects, with only partial overlap between different stimuli (**Figures 4**, **5**; **Supplementary Table 4**). False discovery rate correction gave ‘q’ values higher than 0.05. Therefore, we consider these results as an initial discovery phase due to the small sample size of this rare disease cohort, which will require future replication in other patient cohorts, but may orient future investigations and we believe are worthwhile to discuss here. Interestingly, non-stimulated cells already displayed 20 differentially expressed genes, suggesting some basal changes in circulating immune cells in FAP subjects. Moreover, each of the stimulation conditions revealed several overexpressed genes by FAP subjects. For instance, some MHC class II genes (i.e., HLA-DRA, -DRB3 and -DOB) and IL2RA, which are activation markers in T lymphocytes, were found overexpressed in CD3-CD28-stimulated cells from FAP subjects (**Figure 5B**). Moreover, ICOSLG was found overexpressed in non-stimulated and *Candida*-stimulated cells (**Figure 4B**). ICOS ligand is a costimulatory molecule of the B7 family that when expressed on antigen-presenting cells binds to ICOS, CD28 and CTLA-4 in human T cells. This pathway is involved in T cell activation, differentiation, and production of cytokines, as well as in T cell-mediated B cell responses. ICOSLG/ICOS deficiencies have been associated with immunodeficiency, allergy, inflammation and autoimmunity (38). In the same line of checkpoint inhibitors, LAG-3 (Lymphocyte activation gene 3), coding for an inhibitory protein expressed in activated T cells and a therapeutic target for immunotherapy in cancer (39), was upregulated in non-stimulated, and in *Candida* and CD3-CD28 stimulated cells (**Figures 4A, B**, **5B**). An additional interesting finding is that three key activation molecules in B cells, CD19, CD22 and CD40 (40), were upregulated under several stimulation conditions (**Figures 4A**; **5A, B**). These differences could affect immune response equilibrium in FAP subjects and be related with the increased percentage of B cells (CD19+) (**Figure 3**). Upregulation data would be consistent with a state of basal activation or memory-like functional state in circulating T cells. Several other genes were found downregulated in SEB- and CD3/CD28-stimulated samples of FAP subjects, including CXCR4, a receptor for CXCL12, and CXCR2 (**Figure 5B**). This finding raises the possibility that reduced responsiveness to CXCL12 might account for the impaired migration of FAP subjects’ T cells shown in **Figure 9**. However, our previous findings showing that APC-silenced T cells lines showed similar levels of CXCR4 protein expression (17) and the fact that T cells used for migration assays here were not TCR-CD3 stimulated would not support this hypothesis. Moreover, the expression of MAPK1 gene induced by CD3-CD28 stimulation was inhibited in FAP subjects’ cells. ERK2/MAPK1 is a key signaling serine-threonine kinase that integrates T cell signaling pathways controlling T cell growth and differentiation and the production of cytokines (41). ERK2/MAPK1 is also activated downstream of chemokine receptor stimulation, including CXCL12 (42) and may account for altered T cell migration. Finally, activating/inhibitory KIR genes and FKBP5 were found downregulated under most of the stimulation conditions (**Figure 4**) Altogether, these results suggest that FAP subjects may undergo unbalanced immune responses.

Interestingly, all secreted proteins differentially produced by FAP subjects’ cells, revealed by the Luminex assay, were upregulated, mostly under CD3-CD28 stimulation (**Figure 6**). This included several cytokines, as IFN-α, IFN-γ, TGF-α, IL-1α, IL-15 and IL-33, which are produced by different immune cells. These findings are in line with a memory-like activation state responsible of enhancing some T cell responses. However, in *in vitro* assays, the production of IL-2, IFN-γ and TNF cytokines by PBMC in response to CD3+CD28 stimulation was not significantly different, except for the enhanced proportion of IFNγ restimulated CD8 T cells. Therefore, PBMC activation *in vitro* was less sensitive than whole blood TruCulture-Luminex approach to detect differential cytokine production. Indeed, the latter revealed significant differences in 13 secreted proteins, including 7 cytokines, which may reflect the richer cell environment of whole blood preparations and the multiplex approach. Regarding IFN-γ, both assays showed an upregulated response by cells from FAP subjects (**Figures 6C**, **7D**), suggesting that CD8+ T cells are more affected. Interestingly, IFN-γ could have a dual function either mediating anti-tumor activities or promoting the proliferation of cancer cells (43). IFN-γ contributes to maintain low levels of local chronic inflammation, which could induce genetic modifications favoring the progression to cancer. Moreover, IFN-γ activity on stroma cells may drive more inflammation given the local conditions to predispose the expansion of cancer (44). These findings are in contrast with our previous observations showing that IL-2 expression was inhibited in APC-silenced Jurkat or primary human T cells from healthy subjects (15). This suggests that the potential impact of the APC mutation on cytokine production may be different in T cells from FAP subjects than in APC-silenced cells. Moreover, the results shown here differ from those we obtained in lamina propria T cells from *Apc^Min/+^*, in which several cytokines were inhibited, particularly IL-10 (15). The environment in which lamina propria T cells are embedded may explain the differences with peripheral blood T cells, although they could also be accounted for by differences between the mouse mutant model and FAP subjects. It will be therefore important to investigate intestinal T cell responses in FAP subjects, though much more challenging.

Hence, whole blood approaches allowed us to pinpoint some differences between FAP and healthy subjects. We could not, however, unveil differences between the two groups of FAP subjects expressing or not truncated APC protein. Although needing further investigation, our findings suggest that a dominant negative effect of truncated APC protein would not be the main cause of the differential responses of FAP subjects described here.

To get a deeper insight into the effects observed in FAP subjects, we investigated additional immune response features in purified T cells or in *in vitro* differentiated CTLs, following from our previous observations from APC-silenced cells (15, 16). Cells from FAP and healthy subjects did not show significant differences in *in vitro* T cell proliferation, or expression of differentiation or activation markers (**Figure 8**; **Supplementary Figure 14**). This suggests that the Wnt/β-catenin signaling pathway impaired by APC mutations in intestinal epithelial cells and the potential main cause of dysplasia is not affecting similar processes in *in vitro* stimulated T cells.

Our previous data in APC-silenced *in vitro* differentiated CTLs showed a mild but significant inhibition in their capacity to kill tumor target cells (16). However, CTLs from FAP subjects did not show significant differences but a mild increase in cytotoxicity, with no significant change in degranulation either (**Figure 9**). This might be consistent with an increased production of granzyme B detected in the secreted protein assay. Hence, APC mutations seem to have a lesser effect in T cell-induced killing that the strong reduction in APC expression induced by RNA-interference, at least under the experimental conditions we used. Worth noting, *in vitro* differentiated CTLs are very efficient to kill target cells and their culture in IL2 may overcome genetic defects affecting CTL function through overexpression of other proteins (45). This could counteract the potential effect of APC mutation in cytotoxicity. It is also possible that the anti-CD3-coated P815 target cells in our experiments provide a too strong stimulus preventing the detection of differences between FAP and healthy subject samples. It would be interesting to reduce the E/T ratio and the concentration of anti-CD3 and set the cells in flat bottom plates to try to mimic weak T cell stimulation *in vivo*. Responses might be affected, in this case, by subtle defects in cell migration. However, the variability of responses between subjects might also increase under these conditions, making interpretation more difficult. This was the case when we tried to perform these experiments under live imaging conditions (e.g. Incucyte^®^ technology).

In our previous work, we observed that in APC-silenced CTLs the organization of the actin and microtubule cytoskeleton at the immunological synapse was altered (15, 16). Moreover, immunological synapses of APC-silenced CTLs with target cells were more often dissymmetric, with more irregular shape and lower stability (16). Here, we have developed computer-based methods to measure changes on microtubule organization and in T cell morphology more accurately. In line with our previous results (16), we report here that CTLs from FAP subjects display less organized and more variable microtubule patterns as well (**Figures 10A-C**). Moreover, differences in CTL morphology at the synapse with tumor cells were also observed (**Figures 10G-J**), which could be suggestive of the lower synapse stability in APC-silenced cells that we observed before (16). Finally, F-actin reorganization was less efficient in FAP T cells (**Figures 10D-F**). However, centrosome polarization to the synapse was highly variable and no significant differences could be appreciated.

Therefore, T cells from FAP subjects display several impaired features of immunological synapses reminiscent of those previously observed in APC-silenced T cells (16). However, the differences observed between CTLs from FAP and healthy subjects were less obvious, as a consequence of higher variability.

Interestingly, the most consistent and significant difference between T cells from FAP and healthy subjects was the impaired capacity of FAP subjects’ T cells to migrate through micropores in response to CXCL12 (**Figure 11**). The data reported here enlarge our previous study that included a first group of 7 FAP and healthy subjects, in which we investigated more in depth the molecular mechanism involved in the differences between FAP and healthy subjects and compared them with APC-silenced T cells (17). From that work, we proposed that APC defects impaired the interplay between cytoskeleton and adhesion processes that control T cell migration in constrained environments. These findings are compatible with an impact of APC mutations in the process of tumor infiltration by T cells and underscore the importance of APC as cell polarity regulator in T cell functions and their alteration in FAP subjects (17).

In sum, this study reveals that several immune cell processes might be unbalanced as a potential result of APC mutations in FAP subjects. Together they might result in less efficient antitumor T cell responses. To our knowledge, immune pathologies (e.g., recurrent infections, allergy, autoimmunity) have not been strongly associated with FAP harboring APC mutations. This is consistent with the mild differences observed here in peripheral blood cell responses between FAP and healthy subjects. However, several subtle alterations may add their effects at the tumor site during the long period of polyposis development, diminishing antitumor immunity in FAP subjects. Our study opens new avenues of investigation involving immune responses as additional parameters in APC-mediated polyposis pathology.

## Data availability statement

The datasets presented in this study can be found in online repositories. The names of the repository/repositories and accession number(s) can be found here: EGAS00001007237 (European Genome-phenome Archive).

## Ethics statement

The studies involving human participants were reviewed and approved by Comité de Protection des Personnes, Île de France-1. Protocol N° 2010-déc. 12483 for healthy subjects and N° 2018-mai-14852 for FAP subjects. The patients/participants provided their written informed consent to participate in this study.

## Author contributions

CC: designed methodology, managed sample collection-storage, designed-performed most experiments, analyzed-interpreted-managed data, wrote manuscript. MMas and MJ: designed-performed most experiments, analyzed-interpreted data, edited manuscript. HL and M-NU: designed clinical research protocols, wrote-submitted ethical project, subject information and consent forms, interviewed, informed, advised, performed medical consultations for participants, analyzed medical criteria, withdrew blood samples, analyzed data and edited the manuscript. DK and MIG: developed computer-based image analysis methods, performed image analyses and edited the manuscript. DS-A: designed, analyzed experiments, interpreted data, edited the manuscript. MMad: performed some experiments, analyzed data. SG, CF, GC-LF and PL: designed, performed experiments to generate VHH nanobodies, edited the manuscript. CR: interviewed, informed, advised and performed the medical consultation for participants, analyzed medical criteria, withdrew blood samples, edited the manuscript. MB and CM: advised on clinical protocol and subject information and consent forms, communicated on the project to participants and public, helped FAP subject recruitment, edited manuscript. SS and SN: designed FACS panels and protocols, advised on FACS data acquisition-analysis. SM: advised on data analysis and statistics. MH and DD: advised on whole blood-based approaches, data analysis and interpretation, edited the manuscript. VB: conceived the general project, designed-performed experiments, analyzed-interpreted data, wrote manuscript. AA: conceived the general project and translational research protocols, interpreted data, ensured communication with participants and doctors, coordinated funding, wrote manuscript. All authors contributed to the article and approved the submitted version.
