# Supplementary material for: T cell migration and effector function differences in familial adenomatous polyposis patients with APC gene mutations
Source: Front Immunol. 2023 Jul 18;14:1163466. doi: 10.3389/fimmu.2023.1163466 (PMC10393261; doi:10.3389/fimmu.2023.1163466)
Supplement: Supplementary file 1 [file DataSheet_1.pdf]

Supplementary Material

T cell migration and effector function differences in familial adenomatous polyposis patients with APC gene mutations

Céline Cuche[1], Marta Mastrogiovanni[1,2], Marie Juzans[1,2], Hélène Laude[3], Marie-Noëlle Ungeheuer[3], Maria Isabella Gariboldi[4#], Daniel Krentzel[4#], , Daniel Scott-Algara[1], Marianne Madec[1], Sophie Goyard[1,5], Camille Floch[1,5], Gaëlle Chauveau-Le Friec[6] Pierre Lafaye[6], Charlotte Renaudat[3], Muriel Le Bidan[7], Christine Micallef[7], Sandrine Schmutz[8], Sébastien Mella[8,9], Sophie Novault[8], Milena Hasan[8], Darragh Duffy[10], Vincenzo Di Bartolo[1*†], Andrés Alcover[1*†]

[1] Institut Pasteur, Université Paris Cité, INSERM-U1224, Unité Biologie Cellulaire des Lymphocytes, Ligue Nationale Contre le Cancer-Équipe Labellisée Ligue 2018. Paris, France.
[2] Sorbonne Université, Collège Doctoral, Paris, France.
[3] Institut Pasteur, Université Paris Cité, ICAReB, Paris, France.
[4] Institut Pasteur, Université Paris Cité, CNRS-UMR3691, Unité Imagerie et Modélisation. Paris, France.
[5] Institut Pasteur, Université Paris Cité, Plateforme d'Innovation et de Développement de Tests Diagnostiques. Paris, France.
[6] Institut Pasteur, Université Paris Cité, CNRS-UMR3528, Plateforme d'Ingénierie des Anticorps. Paris, France.
[7] Association Polyposes Familiales France. Linas, France
[8] Institut Pasteur, Université Paris Cité, Unité de Technologie et Service Cytométrie et Biomarqueurs, Paris, France.
[9] Institut Pasteur, Université Paris Cité, Hub Bioinformatique et Biostatistique. Paris, France.
[10] Institut Pasteur, Université Paris Cité, Unité Immunologie Translationnelle. Paris, France.
*Corresponding authors
†VDB and AA share senior authorship
[#] MIG and DK contributed equally to this work
Correspondence: Andres Alcover andres.alcover@pasteur.fr and Vincenzo Di Bartolo vincenzo.di-bartolo@pasteur.fr . Institut Pasteur. 28, Rue du Dr Roux. 75015 Paris. France.

Cell surface marker	Fluorochrome	Clone	Reference	Supplier
CD66b	FITC	G10F5	2125515	Sony
CD25	PE	BC96	302606	Biolegend
HLA-DR	PE-Dazzle594	L243	2138265	Sony
CD45RA	BV570		304132	Biolegend
CD11c	PECy7		301607	Biolegend
CD3	PECy5		555341	Becton Dickinson
CD14	AF532	61D3	58-0149-41	Invitrogen
CD45	BV480	HI30	566115	Becton Dickinson
CD16	BV510	3G8	2110235	Sony
CD8a	BB700	RPA-T8	566451	Becton Dickinson
CD123	BV605	6H6	306025	Biolegend
CD27	BV650	O323	302827	Biolegend
CD56	BV711	HCD56	318336	Biolegend
CD19	BV750	HIB19	302261	Biolegend
TCRgd	BV785	B1	740995	Becton Dickinson
CD4	APC	OKT4	317415	Biolegend
CD3	ECD	UCHT1	A07748	Beckman
CD69	BV785	FN50	310931	Biolegend
CD158d	PE	mAb33	347005	Biolegend
NKG2C	BV605	134591	748166	Becton Dickinson
NKG2D	FITC	1D11	820819	Biolegend
CD85j	PEcy7	GHI/75	333711	Biolegend
CD226 (DNAM)	BV785	DX11	742497	Becton Dickinson
CD161	PECy7	HP-3G10	339917	Biolegend
NKp44 (CD336)	BV605	p44-8	744301	Becton Dickinson
NKp80	PE	5D12	346706	Biolegend
CD158e1 (KIR3DL1)	BV785	CD158e1	742982	Becton Dickinson
NKp46	FITC	9E2	331922	Biolegend
CD158e1/e2 (KIR3DL1/DS1)	PE	Z27.3.7	IM3292	Beckman
NKG2a (CD159a)	BV605	131411	747921	Becton Dickinson
CD160	PECy7	BY55	341211	Biolegend
CD158a (KIR2DL1)	FITC	HP-3E4	556062	Becton Dickinson
2B4 (CD244)	PECy7	C1.7	329520	Biolegend
CD158i	PE	jjC11.6	130-099-698	Miltenyi
CD158b (KIR2DL2/3)	BV605	CH-L	743453	Becton Dickinson
NKp30	BV785	p30-15	743172	Becton Dickinson
NKT	BV480	6B11	746788	Becton Dickinson
Va7.2	BB700	OF-5A12	749483	Becton Dickinson
TCRgd	BV650	B1	564156	Becton Dickinson
CD8a	BV570	RPA-T8	301038	Biolegend

Supplementary Table 1. Whole blood immunophenotyping antibodies

Probe name and NS probe ID Nanostring

probe name	NS Probe ID	probe name	NS Probe ID	probe name	NS Probe ID
ABCB1	NM_000927.3:3910	DPP4	NM_001935.3:2700	LILRA5	NM_181879.2:545
ABL1	NM_005157.3:3200	DUSP4	NM_057158.2:3115	LILRA6	NM_024318.2:1900
ADA	NM_000022.2:1300	EBI3	NM_005755.2:485	LILRB1	NM_001081637.1:2332
AHR	NM_001621.3:1900	EDNRB	NM_003991.2:560	LILRB2	NM_005874.1:595
AICDA	NM_020661.1:567	EGR1	NM_001964.2:1505	LILRB3	NM_006864.2:2235
AIRE	NM_000383.2:1864	EGR2	NM_000399.3:1891	LILRB4	NM_001081438.1:1825
APP	NM_000484.3:1725	ENTPD1	NM_001098175.1:8830	LILRB5	NM_001081442.1:327
ARG1	NM_000045.2:505	EOMES	NM_005442.2:1670	LITAF	NM_004862.3:1000
ARG2	NM_001172.3:1150	ETS1	NM_005238.3:4625	LTA	NM_000595.2:885
ARHGDIB	NM_001175.4:833	FADD	NM_003824.2:1560	LTB4R	NM_181657.3:2400
ATG10	NM_001131028.1:985	FAS	NM_000043.3:90	LTB4R2	NM_019839.4:1250
ATG12	NM_004707.2:25	FCAR	NM_133280.1:75	LTBR	NM_002342.1:1435
ATG16L1	NM_198890.2:1975	FCER1A	NM_002001.2:114	LTF	NM_002343.2:590
ATG5	NM_004849.2:1104	FCER1G	NM_004106.1:36	LY96	NM_015364.2:360
ATG7	NM_001136031.2:810	FCGR1A/B	NM_000566.3:1545	MAF	NM_005360.4:2198
ATM	NM_000051.3:30	FCGR2A	NM_021642.3:60	MALT1	NM_006785.2:909
B2M	NM_004048.2:235	FCGR2A/C	NM_201563.4:1166	MAP4K1	NM_007181.3:780
B3GAT1	NM_018644.3:2388	FCGR2B	NM_001002273.1:870	MAP4K2	NM_004579.2:1610
BATF	NM_006399.3:293	FCGR3A/B	NM_000570.4:696	MAP4K4	NM_004834.3:3315
BATF3	NM_018664.2:770	FCGRT	NM_004107.4:1276	MAPK1	NM_138957.2:430
BAX	NM_138761.3:342	FKBP5	NM_001145775.1:540	MAPK11	NM_002751.5:1310
BCAP31	NM_005745.7:495	FN1	NM_212482.1:1776	MAPK14	NM_001315.1:450
BCL10	NM_003921.2:1250	FOXP3	NM_014009.3:1230	MAPKAPK2	NM_004759.3:710
BCL2	NM_000657.2:947	FYN	NM_002037.3:765	MARCO	NM_006770.3:1434
BCL2L11	NM_138621.4:257	GATA3	NM_001002295.1:2835	MASP1	NM_139125.3:35
BCL3	NM_005178.2:450	GBP1	NM_002053.1:2110	MASP2	NM_139208.1:330
BCL6	NM_001706.2:675	GBP5	NM_052942.3:1955	MBL2	NM_000242.2:1756
BID	NM_001196.2:1875	GFI1	NM_005263.2:2235	MBP	NM_002385.2:132
BLNK	NM_013314.2:930	GNLY	NM_006433.2:305	MCL1	NM_021960.3:1260
BST1	NM_004334.2:710	GP1BB	NM_000407.4:146	MIF	NM_002415.1:319
BST2	NM_004335.2:560	GPI	NM_000175.2:1695	MME	NM_000902.2:5059
BTK	NM_000061.1:570	GPR183	NM_004951.3:405	MR1	NM_001531.2:7695
BTLA	NM_181780.2:305	GZMA	NM_006144.2:155	MRC1	NM_002438.2:525
C14orf166	NM_016039.2:210	GZMB	NM_004131.3:540	MS4A1	NM_152866.2:620
C1QA	NM_015991.2:718	GZMK	NM_002104.2:700	MSR1	NM_002445.3:326
C1QB	NM_000491.3:819	HAMP	NM_021175.2:99	MUC1	NM_001018017.1:725
C1QBP	NM_001212.3:745	HAVCR2	NM_032782.3:955	MX1	NM_002462.2:1485
C1R	NM_001733.4:760	HFE	NM_139011.2:875	MYD88	NM_002468.3:2145
C1S	NM_001734.2:775	HLA-A	NM_002116.5:1000	NCAM1	NM_000615.5:1620
C2	NM_000063.3:1075	HLA-B	NM_005514.6:937	NCF4	NM_000631.4:210
C3	NM_000064.2:4396	HLA-C	NM_002117.4:895	NCR1	NM_004829.5:602
C4A/B	NM_007293.2:1217	HLA-DMA	NM_006120.3:380	NFATC1	NM_172389.1:1984
C4BPA	NM_000715.3:690	HLA-DMB	NM_002118.3:20	NFATC2	NM_012340.3:1815
C5	NM_001735.2:2592	HLA-DOB	NM_002120.3:230	NFATC3	NM_004555.2:2190
C6	NM_000065.2:3170	HLA-DPA1	NM_033554.2:857	NFIL3	NM_005384.2:1795
C7	NM_000587.2:310	HLA-DPB1	NM_002121.4:931	NFKB1	NM_003998.2:1675
C8A	NM_000562.2:690	HLA-DQA1	NM_002122.3:261	NFKB2	NM_002502.2:825
C8B	NM_000066.2:620	HLA-DQB1	NM_002123.3:384	NFKBIA	NM_020529.1:945
C8G	NM_000606.2:407	HLA-DRA	NM_019111.3:335	NFKBIZ	NM_001005474.1:2030
C9	NM_001737.3:602	HLA-DRB1	NM_002124.2:104	NLRP3	NM_001079821.2:415
CAMP	NM_004345.3:220	HLA-DRB3	NM_022555.3:698	NOD1	NM_006092.1:3285
CARD9	NM_052813.4:1525	HRAS	NM_005343.2:396	NOD2	NM_022162.1:4080
CASP1	NM_001223.3:971	ICAM1	NM_000201.2:2253	NOS2	NM_000625.4:605
CASP10	NM_032977.3:20	ICAM2	NM_000873.3:415	NOTCH1	NM_017617.3:735
CASP2	NM_032982.2:1710	ICAM3	NM_002162.3:1225	NOTCH2	NM_024408.3:2842

Supplementary Table 2.1. Expressed genes assessed by Nanostring (1/4)

Probe name and NS probe ID Nanostring

probe name	NS Probe ID	probe name	NS Probe ID	probe name	NS Probe ID
CASP3	NM_032991.2:685	ICAM4	NM_001039132.1:463	NT5E	NM_002526.2:1214
CASP8	NM_001228.4:301	ICAM5	NM_003259.3:327	PAX5	NM_016734.1:2288
CCBP2	NM_001296.3:1345	ICOS	NM_012092.2:640	PDCD1	NM_005018.1:175
CCL11	NM_002986.2:378	ICOSLG	NM_015259.4:1190	PDCD1LG2	NM_025239.3:235
CCL13	NM_005408.2:320	IDO1	NM_002164.3:50	PDCD2	NM_144781.2:288
CCL15	NM_032965.3:112	IFI16	NM_005531.1:2255	PDGFB	NM_033016.2:1480
CCL16	NM_004590.2:367	IFI35	NM_005533.3:415	PDGFRB	NM_002609.3:840
CCL18	NM_002988.2:585	IFIH1	NM_022168.2:185	PECAM1	NM_000442.3:1365
CCL19	NM_006274.2:401	IFIT2	NM_001547.4:1995	PIGR	NM_002644.2:215
CCL2	NM_002982.3:123	IFITM1	NM_003641.3:482	PLA2G2A	NM_000300.2:715
CCL20	NM_004591.1:35	IFNA1/13	NM_024013.1:585	PLA2G2E	NM_014589.1:266
CCL22	NM_002990.3:797	IFNA2	NM_000605.3:611	PLAU	NM_002658.2:793
CCL23	NM_145898.1:336	IFNAR1	NM_000629.2:3123	PLAUR	NM_001005376.1:440
CCL24	NM_002991.2:18	IFNAR2	NM_000874.3:631	PML	NM_002675.3:281
CCL26	NM_006072.4:184	IFNB1	NM_002176.2:610	POU2F2	NM_002698.2:908
CCL3	NM_002983.2:159	IFNG	NM_000619.2:970	PPARG	NM_015869.3:1035
CCL4	NM_002984.2:35	IFNGR1	NM_000416.1:1140	PPBP	NM_002704.2:330
CCL5	NM_002985.2:280	IGF2R	NM_000876.1:2605	PRDM1	NM_001198.3:798
CCL7	NM_006273.2:120	IKBKAP	NM_003640.3:1130	PRF1	NM_005041.3:2120
CCL8	NM_005623.2:689	IKBKB	NM_001556.1:1995	PRKCD	NM_006254.3:2165
CCND3	NM_001760.2:1215	IKBKE	NM_014002.2:2470	PSMB10	NM_002801.2:221
CCR1	NM_001295.2:535	IKBKG	NM_003639.2:470	PSMB5	NM_001130725.1:716
CCR10	NM_016602.2:843	IKZF1	NM_006060.3:4485	PSMB7	NM_002799.2:420
CCR2	NM_001123041.2:743	IKZF2	NM_016260.2:870	PSMB8	NM_004159.4:1215
CCR5	NM_000579.1:2730	IKZF3	NM_183232.2:1176	PSMB9	NM_002800.4:455
CCR6	NM_031409.2:935	IL10	NM_000572.2:230	PSMC2	NM_002803.3:446
CCR7	NM_001838.2:1610	IL10RA	NM_001558.2:150	PSMD7	NM_002811.3:580
CCR8	NM_005201.2:245	IL11RA	NM_147162.1:400	PTAFR	NM_000952.3:1035
CCRL1	NM_016557.2:854	IL12A	NM_000882.2:775	PTGER4	NM_000958.2:1380
CCRL2	NM_003965.4:1110	IL12B	NM_002187.2:1435	PTGS2	NM_000963.1:495
CD14	NM_000591.2:885	IL12RB1	NM_005535.1:225	PTK2	NM_005607.3:1005
CD160	NM_007053.2:500	IL13	NM_002188.2:516	PTPN2	NM_002828.2:185
CD163	NM_004244.4:1630	IL13RA1	NM_001560.2:1230	PTPN22	NM_015967.4:2505
CD164	NM_006016.4:2575	IL15	NM_172174.1:1685	PTPN6	NM_002831.5:1734
CD19	NM_001770.4:1770	IL16	NM_004513.4:1262	PTPRC_all	NM_080921.2:90
CD1A	NM_001763.2:1815	IL17A	NM_002190.2:240	PYCARD	NM_013258.3:714
CD1D	NM_001766.3:1428	IL17B	NM_014443.2:177	RAF1	NM_002880.2:1990
CD2	NM_001767.3:687	IL17F	NM_052872.3:210	RAG1	NM_000448.2:2300
CD209	NM_021155.2:1532	IL18	NM_001562.2:48	RAG2	NM_000536.3:655
CD22	NM_001771.2:2515	IL18R1	NM_003855.2:2025	RARRES3	NM_004585.3:640
CD24	NM_013230.2:95	IL18RAP	NM_003853.2:2412	RELA	NM_021975.2:360
CD244	NM_016382.2:1150	IL19	NM_013371.3:1030	RELB	NM_006509.2:250
CD247	NM_198053.1:1490	IL1A	NM_000575.3:1085	RORC	NM_001001523.1:1350
CD27	NM_001242.4:330	IL1B	NM_000576.2:840	RUNX1	NM_001754.4:635
CD274	NM_014143.3:1245	IL1R1	NM_000877.2:4295	S100A8	NM_002964.3:115
CD276	NM_001024736.1:2120	IL1R2	NM_173343.1:113	S100A9	NM_002965.2:75
CD28	NM_001243078.1:2065	IL1RAP	NM_002182.2:460	S1PR1	NM_001400.3:1065
CD34	NM_001025109.1:1580	IL1RL1	NM_016232.4:700	SELE	NM_000450.2:1505
CD36	NM_001001548.2:705	IL1RL2	NM_003854.2:595	SELL	NR_029467.1:1585
CD3D	NM_000732.4:110	IL1RN	NM_000577.3:480	SELPLG	NM_003006.3:2297
CD3E	NM_000733.2:75	IL2	NM_000586.2:300	SERPING1	NM_000062.2:305
CD3EAP	NM_012099.1:555	IL20	NM_018724.3:292	SH2D1A	NM_001114937.2:495
CD4	NM_000616.4:975	IL21	NM_021803.2:65	SIGIRR	NM_021805.2:469
CD40	NM_001250.4:1265	IL21R	NM_021798.2:2080	SKI	NM_003036.2:3335
CD40LG	NM_000074.2:1225	IL22	NM_020525.4:319	SLAMF1	NM_003037.2:580
CD44	NM_001001392.1:429	IL22RA2	NM_181310.1:290	SLAMF6	NM_001184714.1:1032
CD45R0	NM_080921.3:258	IL23A	NM_016584.2:411	SLAMF7	NM_021181.3:215

Supplementary Table 2.2. Expressed genes assessed by Nanostring (2/4)

Probe name and NS probe ID Nanostring

probe name	NS Probe ID	probe name	NS Probe ID	probe name	NS Probe ID
CD45RA	NM_002838.4:258	IL23R	NM_144701.2:710	SLC2A1	NM_006516.2:2500
CD45RB	ENST00000367367.1:131	IL26	NM_018402.1:80	SMAD3	NM_005902.3:4220
CD46	NM_172350.1:365	IL27	NM_145659.3:143	SMAD5	NM_005903.5:1044
CD48	NM_001778.2:270	IL28A	NM_172138.1:589	SOCS1	NM_003745.1:1025
CD5	NM_014207.2:1295	IL28A/B	NM_172139.2:104	SOCS3	NM_003955.3:1870
CD53	NM_001040033.1:835	IL29	NM_172140.1:233	SPP1	NM_000582.2:760
CD55	NM_000574.3:101	IL2RA	NM_000417.1:1000	SRC	NM_005417.3:1410
CD58	NM_001779.2:478	IL2RB	NM_000878.2:1980	STAT1	NM_007315.2:205
CD59	NM_000611.4:730	IL2RG	NM_000206.1:595	STAT2	NM_005419.2:1965
CD6	NM_006725.3:1280	IL3	NM_000588.3:130	STAT3	NM_139276.2:4535
CD7	NM_006137.6:440	IL32	NM_001012633.1:758	STAT4	NM_003151.2:789
CD70	NM_001252.2:190	IL4	NM_000589.2:625	STAT5A	NM_003152.2:3460
CD74	NM_001025159.1:964	IL4R	NM_000418.2:705	STAT5B	NM_012448.3:200
CD79A	NM_001783.3:695	IL5	NM_000879.2:105	STAT6	NM_003153.3:2030
CD79B	NM_021602.2:24	IL6	NM_000600.1:220	SYK	NM_003177.3:1685
CD80	NM_005191.3:1288	IL6R	NM_000565.2:993	TAGAP	NM_054114.3:169
CD81	NM_004356.3:735	IL6ST	NM_002184.2:2505	TAL1	NM_003189.2:4635
CD82	NM_002231.3:1211	IL7	NM_000880.2:38	TAP1	NM_000593.5:2075
CD83	NM_004233.3:1960	IL7R	NM_002185.2:1610	TAP2	NM_000544.3:909
CD86	NM_175862.3:1265	IL8	NM_000584.2:25	TAPBP	NM_003190.4:1536
CD8A	NM_001768.5:1320	IL9	NM_000590.1:300	TBK1	NM_013254.2:1610
CD8B	NM_004931.3:440	ILF3	NM_001137673.1:730	TBX21	NM_013351.1:890
CD9	NM_001769.2:405	IRAK1	NM_001569.3:1995	TCF4	NM_003199.1:1455
CD96	NM_005816.4:1355	IRAK2	NM_001570.3:1285	TCF7	NM_003202.2:2420
CD97	NM_078481.2:1370	IRAK3	NM_007199.1:1735	TFRC	NM_003234.1:1220
CD99	NM_002414.3:625	IRAK4	NM_016123.1:2175	TGFB1	NM_000660.3:1260
CDH5	NM_001795.3:3405	IRF1	NM_002198.1:510	TGFBI	NM_000358.2:2030
CDKN1A	NM_000389.2:1975	IRF3	NM_001571.5:1303	TGFBR1	NM_004612.2:4280
CEACAM1	NM_001712.3:2455	IRF4	NM_002460.1:325	TGFBR2	NM_001024847.1:1760
CEACAM6	NM_002483.4:1217	IRF5	NM_002200.3:1845	THY1	NM_006288.2:135
CEACAM8	NM_001816.3:825	IRF7	NM_001572.3:1763	TICAM1	NM_014261.1:518
CEBPB	NM_005194.2:1420	IRF8	NM_002163.2:253	TIGIT	NM_173799.2:1968
CFB	NM_001710.5:2029	IRGM	NM_001145805.1:1412	TIRAP	NM_148910.2:661
CFD	NM_001928.2:859	ITGA2B	NM_000419.3:740	TLR1	NM_003263.3:545
CFH	NM_001014975.2:702	ITGA4	NM_000885.4:975	TLR2	NM_003264.3:180
CFI	NM_000204.3:1780	ITGA5	NM_002205.2:925	TLR3	NM_003265.2:230
CFP	NM_002621.2:380	ITGA6	NM_000210.1:3065	TLR4	NM_138554.2:2570
CHUK	NM_001278.3:860	ITGAE	NM_002208.4:3405	TLR5	NM_003268.3:215
CIITA	NM_000246.3:470	ITGAL	NM_002209.2:3905	TLR7	NM_016562.3:4120
CISH	NM_145071.2:558	ITGAM	NM_000632.3:515	TLR8	NM_016610.2:2310
CLEC4A	NM_194448.2:388	ITGAX	NM_000887.3:700	TLR9	NM_017442.2:985
CLEC4E	NM_014358.2:570	ITGB1	NM_033666.2:2000	TMEM173	NM_198282.1:725
CLEC5A	NM_013252.2:615	ITGB2	NM_000211.2:520	TNF	NM_000594.2:1010
CLEC6A	NM_001007033.1:342	ITLN1	NM_017625.2:975	TNFAIP3	NM_006290.2:260
CLEC7A	NM_197954.2:55	ITLN2	NM_080878.2:135	TNFAIP6	NM_007115.2:250
CLU	NM_001831.2:2340	JAK1	NM_002227.1:285	TNFRSF10C	NM_003841.3:682
CMKLR1	NM_004072.1:770	JAK2	NM_004972.2:455	TNFRSF11A	NM_003839.2:490
CR1	NM_000651.4:1695	JAK3	NM_000215.2:1715	TNFRSF13B	NM_012452.2:160
CR2	NM_001006658.1:485	KCNJ2	NM_000891.2:620	TNFRSF13C	NM_052945.3:789
CRADD	NM_003805.3:675	KIR3DL1	NM_013289.2:1691	TNFRSF14	NM_003820.2:916
CSF1	NM_000757.4:823	KIR3DL2	NM_006737.2:884	TNFRSF17	NM_001192.2:635
CSF1R	NM_005211.2:3775	KIR3DL3	NM_153443.3:539	TNFRSF1B	NM_001066.2:835
CSF2	NM_000758.2:475	ctivating_Subgr	NM_001083539.1:1146	TNFRSF4	NM_003327.2:200
CSF2RB	NM_000395.2:3300	ctivating_Subgr	NM_014512.1:718	TNFRSF8	NM_152942.2:2030
CSF3R	NM_156038.2:90	nhibiting_Subgr	NM_014218.2:872	TNFRSF9	NM_001561.4:255
CTLA4-TM	NM_005214.3:554	nhibiting_Subgr	NM_014511.3:592	TNFSF10	NM_003810.2:115
CTLA4_all	NM_005214.3:405	KIT	NM_000222.2:2644	TNFSF11	NM_003701.2:490

Supplementary Table 2.3. Expressed genes assessed by Nanostring (3/4)

Probe name and NS probe ID Nanostring

probe name	NS Probe ID	probe name	NS Probe ID	probe name	NS Probe ID
CTNNB1	NM_001098210.1:1815	KLRAP1	NR_028045.1:414	TNFSF12	NM_003809.2:339
CTSC	NM_001814.4:260	KLRB1	NM_002258.2:85	TNFSF13B	NM_006573.4:1430
CTSG	NM_001911.2:160	KLRC1	NM_002259.3:335	TNFSF15	NM_001204344.1:2338
CTSS	NM_004079.3:685	KLRC2	NM_002260.3:942	TNFSF4	NM_003326.2:545
CUL9	NM_015089.2:2675	KLRC3	NM_007333.2:598	TNFSF8	NM_001244.3:518
CX3CL1	NM_002996.3:140	KLRC4	NM_013431.2:276	TOLLIP	NM_019009.2:1320
CX3CR1	NM_001337.3:1040	KLRD1	NM_002262.3:542	TP53	NM_000546.2:1330
CXCL1	NM_001511.1:742	KLRF1	NM_016523.1:275	TRAF1	NM_005658.3:3735
CXCL10	NM_001565.1:40	KLRF2	NM_001190765.1:535	TRAF2	NM_021138.3:1325
CXCL11	NM_005409.4:282	KLRG1	NM_005810.3:65	TRAF3	NM_145725.1:1795
CXCL12	NM_000609.5:210	KLRG2	NM_198508.2:1347	TRAF4	NM_004295.2:1060
CXCL13	NM_006419.2:210	KLRK1	NM_007360.1:760	TRAF5	NM_004619.3:400
CXCL2	NM_002089.3:854	LAG3	NM_002286.5:1735	TRAF6	NM_145803.1:1839
CXCL9	NM_002416.1:1975	LAIR1	NM_002287.3:1195	TYK2	NM_003331.3:485
CXCR1	NM_000634.2:1950	LAMP3	NM_014398.3:1400	UBE2L3	NM_198157.1:990
CXCR2	NM_001557.2:2055	LCK	NM_005356.2:1260	VCAM1	NM_001078.3:2535
CXCR3	NM_001504.1:80	LCP2	NM_005565.3:1210	VTN	NM_000638.3:1346
CXCR4	NM_003467.2:1335	LEF1	NM_016269.3:1165	XBP1	NM_005080.2:440
CXCR6	NM_006564.1:95	LGALS3	NM_001177388.1:495	XCL1	NM_002995.1:620
CYBB	NM_000397.3:2686	LIF	NM_002309.3:1240	XCR1	NM_005283.2:700
DEFB1	NM_005218.3:40	LILRA1	NM_006863.1:1719	ZAP70	NM_001079.3:1175
DEFB103A	NM_001081551.2:106	LILRA2	NM_006866.2:317	ZBTB16	NM_006006.4:1585
DEFB103B	NM_018661.3:114	LILRA3	NM_006865.3:1123	ZEB1	NM_001128128.1:1450
DEFB4A	NM_004942.2:97	LILRA4	NM_012276.3:1577	sCTLA4	NM_001037631.1:560

Supplementary Table 2.4. Expressed genes assessed by Nanostring (4/4)

Luminex Assay – Analytes	
CD40 Ligand	IL-8/CXCL8
EGF	IL-10
Eotaxin/CCL11	IL-12p70
FGF basic	IL-13
Flt-3 ligand	IL-15
G-CSF	IL-17A
GM-CSF	IL-17E/IL-25
Granzyme B	IL-33
GROα/CXCL1	IP-10/CXCL10
GROβ/CXCL2	MCP-1/CCL2
IFNα	MIP-1α/CCL3
IFNβ	MIP-1β/CCL4
IFNγ	MIP-3α/CCL20
IL-1α	MIP-3β/CCL19
IL-1β	PDGF-AA
IL-1ra	PDGF-AB/BB
IL-2	PD-L1/B7-H1
IL-3	RANTES
IL-4	TGF-α
IL-5	TNF
IL-6	TRAIL
IL-7	VEGF

Supplementary Table 3. Secreted proteins assessed by Luminex

Gene\stim	ns	C.alb	SEB	CD3/28	Gene\stim	ns	C.alb	SEB	CD3/28
ABCB1				down*	IL12B		up**	down*	
ATM		up*			IL13RA1				down*
BLNK			up**		IL16	up**			
CAMP			down*	down*	IL22RA2		up*		
CASP3		up*			IL23A		down*		
CASP8				down *	IL23R	up*			
CEACAM6				down *	IRAK1	up*			up**
CEACAM8				down *	IRF5				up*
CCL2		down*			ITLN1				down*
CCL3				up*	JAK1	up*			
CCL7		down**			KCNJ2		up*		
CCR1			up*		KIR_Act_Subgr_1	down*			down*
CCRL1		up*			KIR_Inh_Subgr_1			down*	down*
CD3EAP		down *			KIR_Inh_Subgr_2	down*		down*	down*
CD7			down*		KLRF1			down*	down*
CD8B			down*		LAG3	down*	down*		up*
CD19	up*				LEF1			down*	
CD22		up*	up*	up*	LIF		up*		
CD40				up*	LTF				down*
CD45RB				down*	MALT1			down*	
CD70				up*	MAPK1				down***
CD79B		up*	up*		MAP4K1				up*
CD96			down*		MS4A1	up*	up*	up*	
CFB				up*	NCR1			down*	down*
CFD	up*	up*			NOD1				up*
CFI		up*			NOTCH2		up**		
CFP				down*	PAX5		up*		
CLEC4A			up*		PDCD1				up*
CR2		up*			PECAM1				down*
CSF1	up*	down*			POU2F2		up**	up*	
CTNNB1				down*	POLR2A		up*		
CXCL2				up*	PTGR4				down*
CXCR2				down*	PRF1				down*
CXCR4			down*	down*	PSMB5		down*		
FADD		up*			PTK2	down*			down*
FKBP5	down**	down**	down*	down***	PTPN2	up*			
FOXP3		up*			RUNX1				up*
FYN			down*		SELL				down*
GPR183			down*		SKI		up*		
HAVCR2		down*			TBK1		up*		
HLA-DOB				up*	TCF4	up*			
HLA-DRA				up*	TFRC				down**
HLA-DRB3				up*	TGBR1		up*		
HPRT1	down*		down**		TGFBR2				down*
ICOSLG	up*	up*			TNFRSF11A	down*			
IKZF1				down*	TNFRSF13B	up*			
IL1R2		down*			TNFSF12				up*
IL1RL1	down**		down*		TRAF1				
IL2RA				up*	TRAF6		up*		
IL3		up*			TUBB				up**

Supplementary Table 4. Differentially expressed genes detected by the NanoString analysis. List of differentially expressed genes in FAP *versus* control subjects.
Up: upregulated. Down: downregulated in FAP. *p<0.05 ; **p<0.01 ; ***p<0.001

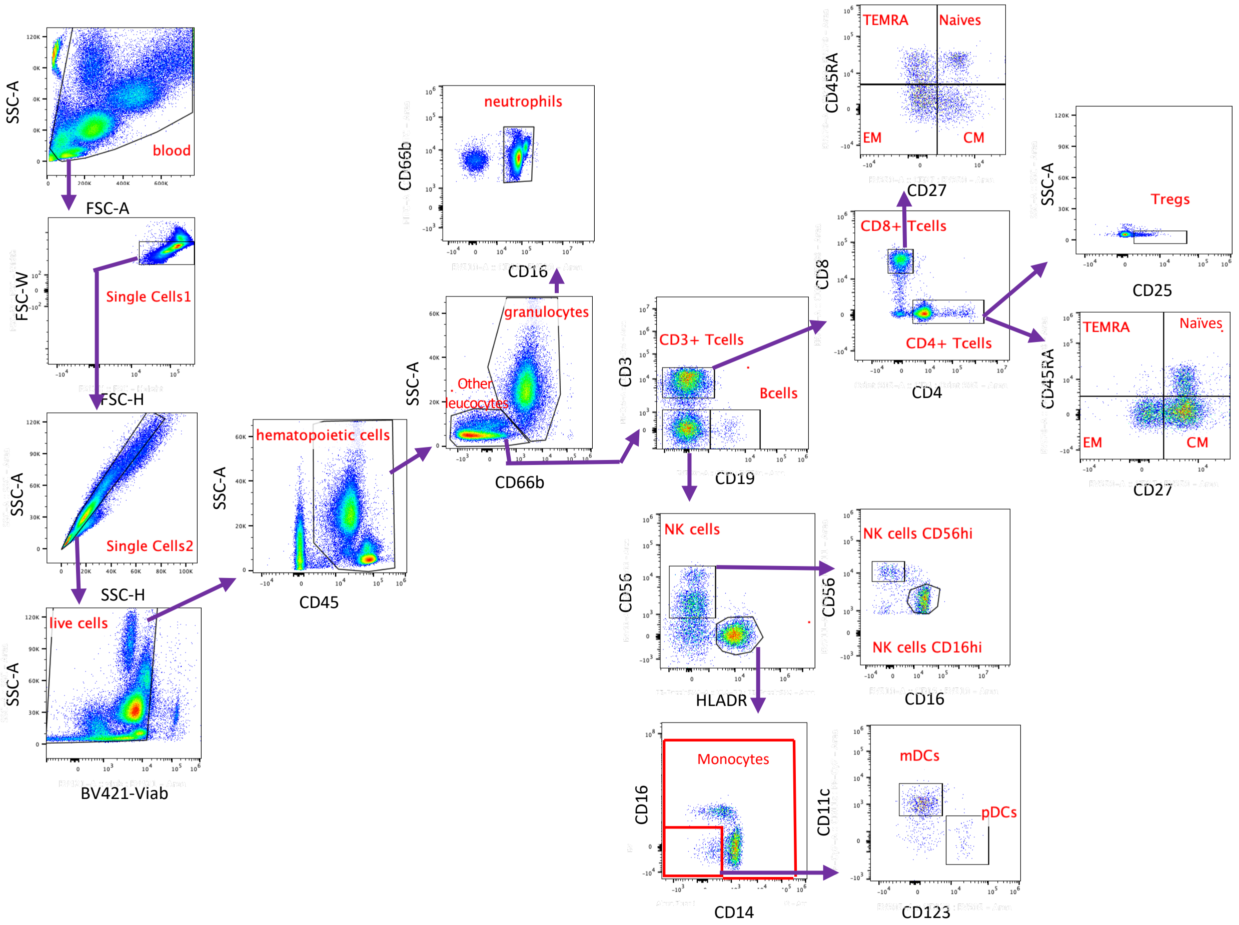

Supplementary Figure 1. **Whole blood immunophenotyping gating strategies**. Whole blood immune cells were stained with directly-coupled fluorescent antibodies (Supplementary Table 1) and analyzed by flow cytometry. The sequential gating strategies leading to the analyses of the main hematopoietic cell populations is depicted.

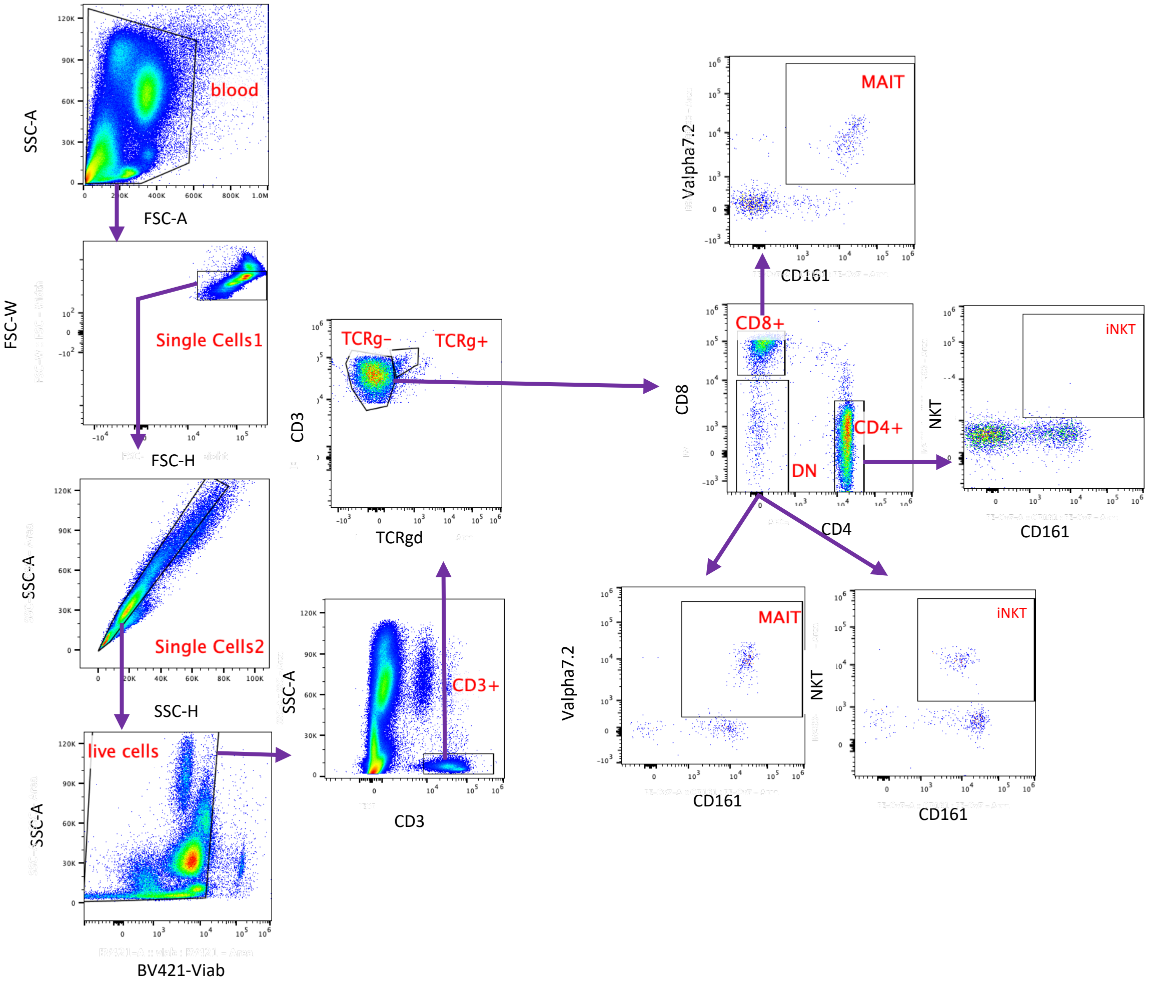

Supplementary Figure 2. **Whole blood immunophenotyping gating strategies**. Whole blood immune cells were stained with directly-coupled fluorescent antibodies (supplementary Table 1) and analyzed by flow cytometry. Sequential gating strategies leading to the analyses of the iNTK; MAIT and TCRγδ T cell populations is depicted.

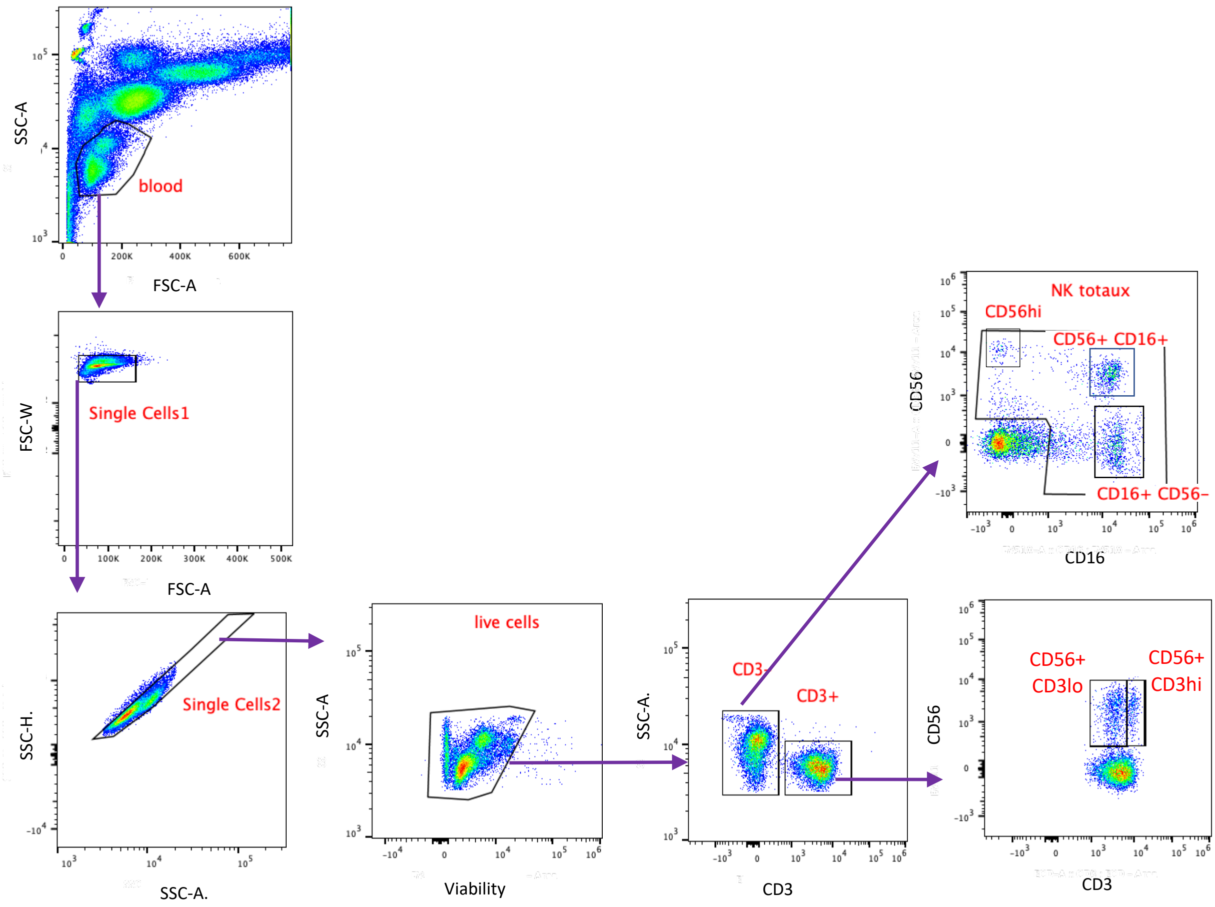

Supplementary Figure 3. **Whole blood immunophenotyping gating strategies**. Whole blood immune cells were stained with directly-coupled fluorescent antibodies (supplementary Table 1) and analyzed by flow cytometry. Sequential gating strategies leading to the analyses of the NK cells subpopulations, is depicted.

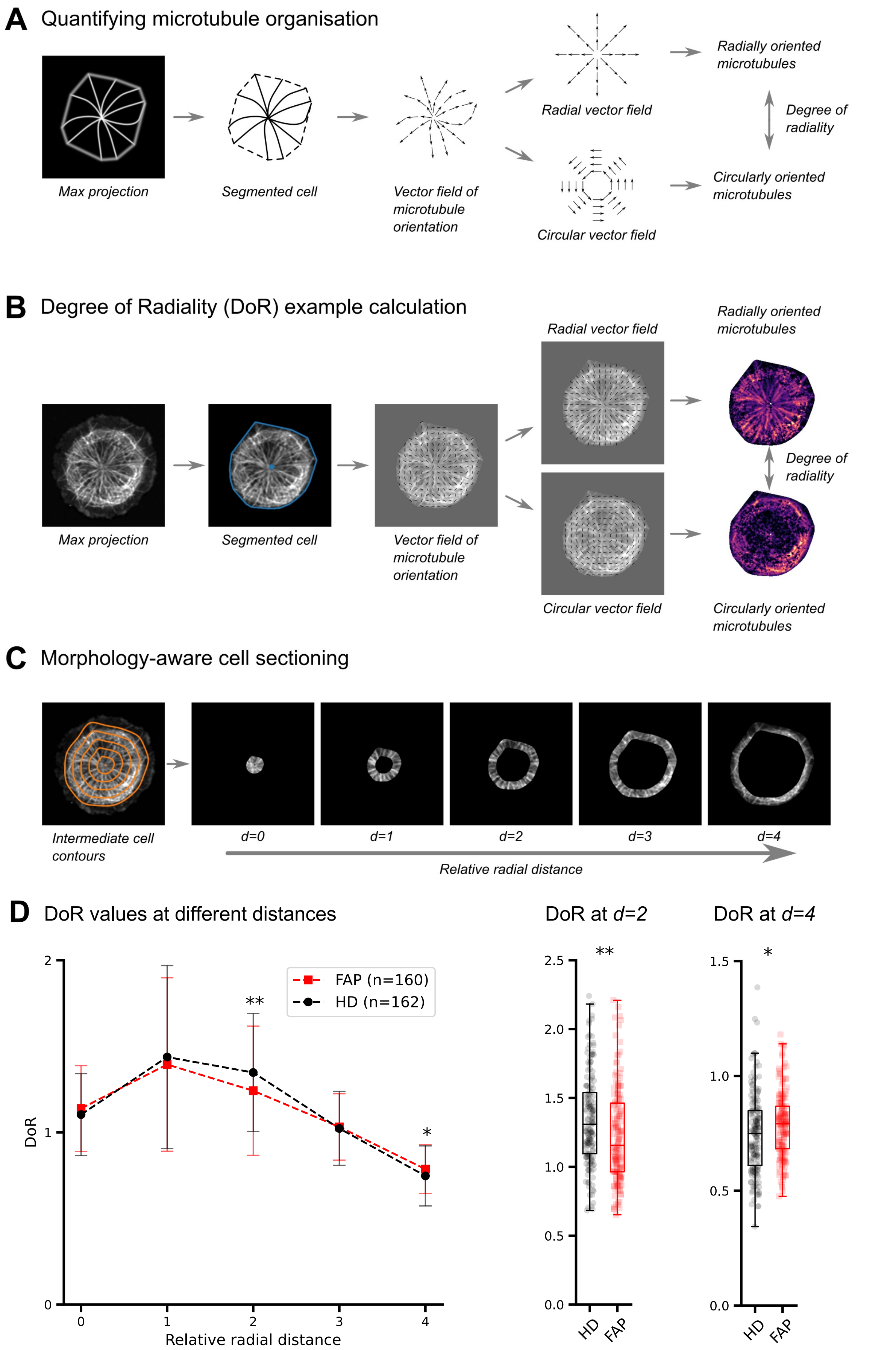

Supplementary Figure 4. **Quantitative analysis of microtubule patterns**. **(A-C)** Schematic representation of the methodology described in the Methods section. **(D)** Quantification of the Degree of Radiality (DoR) as a function of the relative radial distance from the cell center of mass. Data at d=2 (middle panel) are the same shown in Figure 10 C.

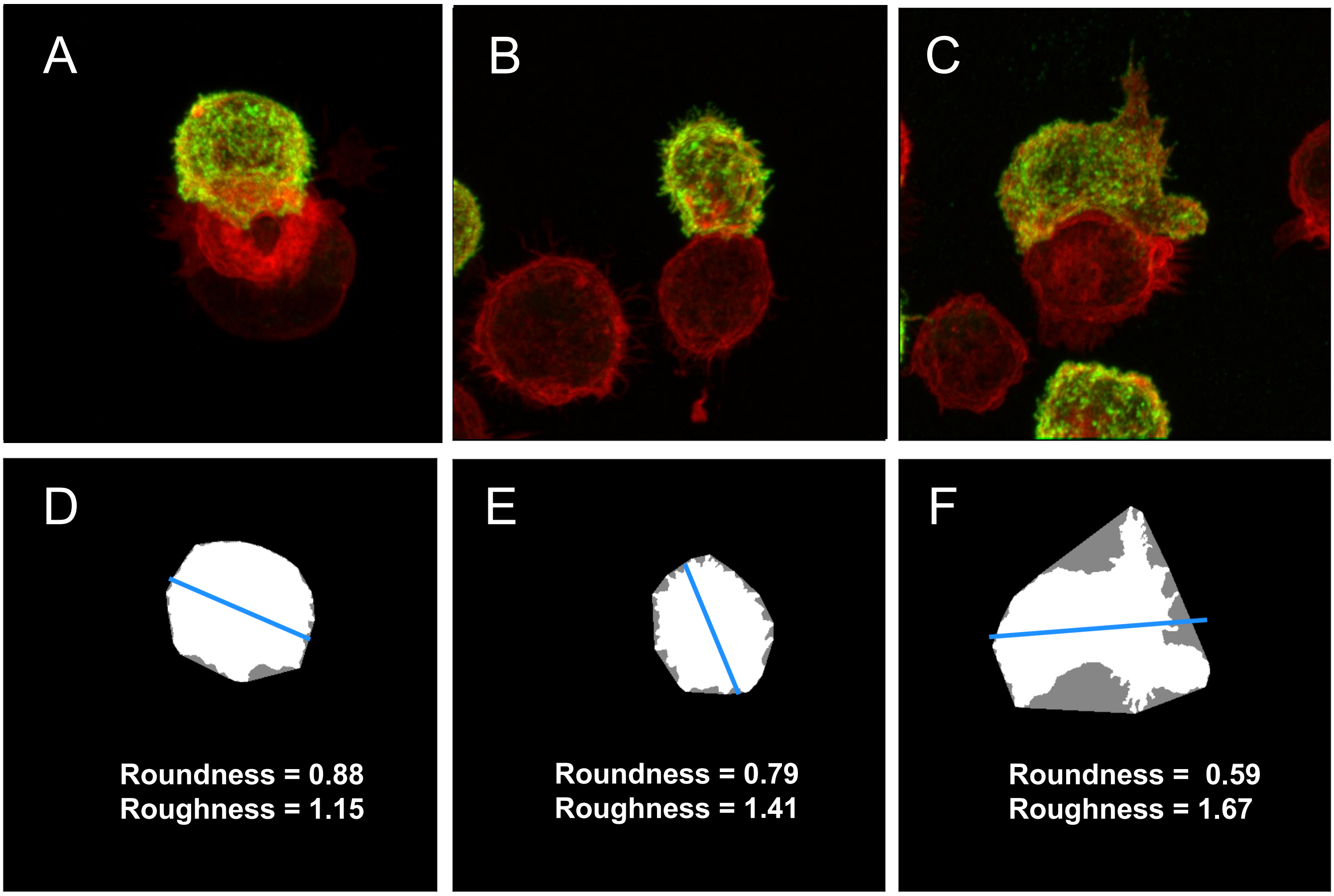

Supplementary Figure 5. **Quantitative analysis of CTL morphology at the immunological synapse.** CTLs engaged on immunological synapses with tumor target cells were prepared, observed and analyzed as described in Methods. Analyses were conducted on maximum intensity projections of confocal microscopy image stacks. (**A-C**) Examples of distinct morphologies observed in T cell–target cell conjugates staining of CD8 (green) and actin (red) for three example cells. **(D-F)** T cell masks generated by thresholding the CD8 signal are displayed in white, overlapped to their convex hull (grey) and the fitted major axis (MA; blue). The roughness of the cell was measured as the ratio of the perimeter of the cell to the perimeter of its convex hull. Higher values indicate higher degrees of cell surface roughness. The roundness was calculated as (4 x T cell mask area)/(π x MA^2). A roundness closer to 1.0 indicates a shape closer to a perfect circle. While the metrics are not completely unrelated, roundness is less sensitive to local irregularities being based on the area and thus can be seen as generally providing information on the cell larger scale morphological irregularity. Roughness, on the other hand, better captures variations in small scale irregularities of the cell surface. The Crofton perimeter was used, which approximates the perimeter based on the Crofton method in four directions. The MA is defined as the length of the major axis of an ellipse with the same normalized second central moment as the mask.

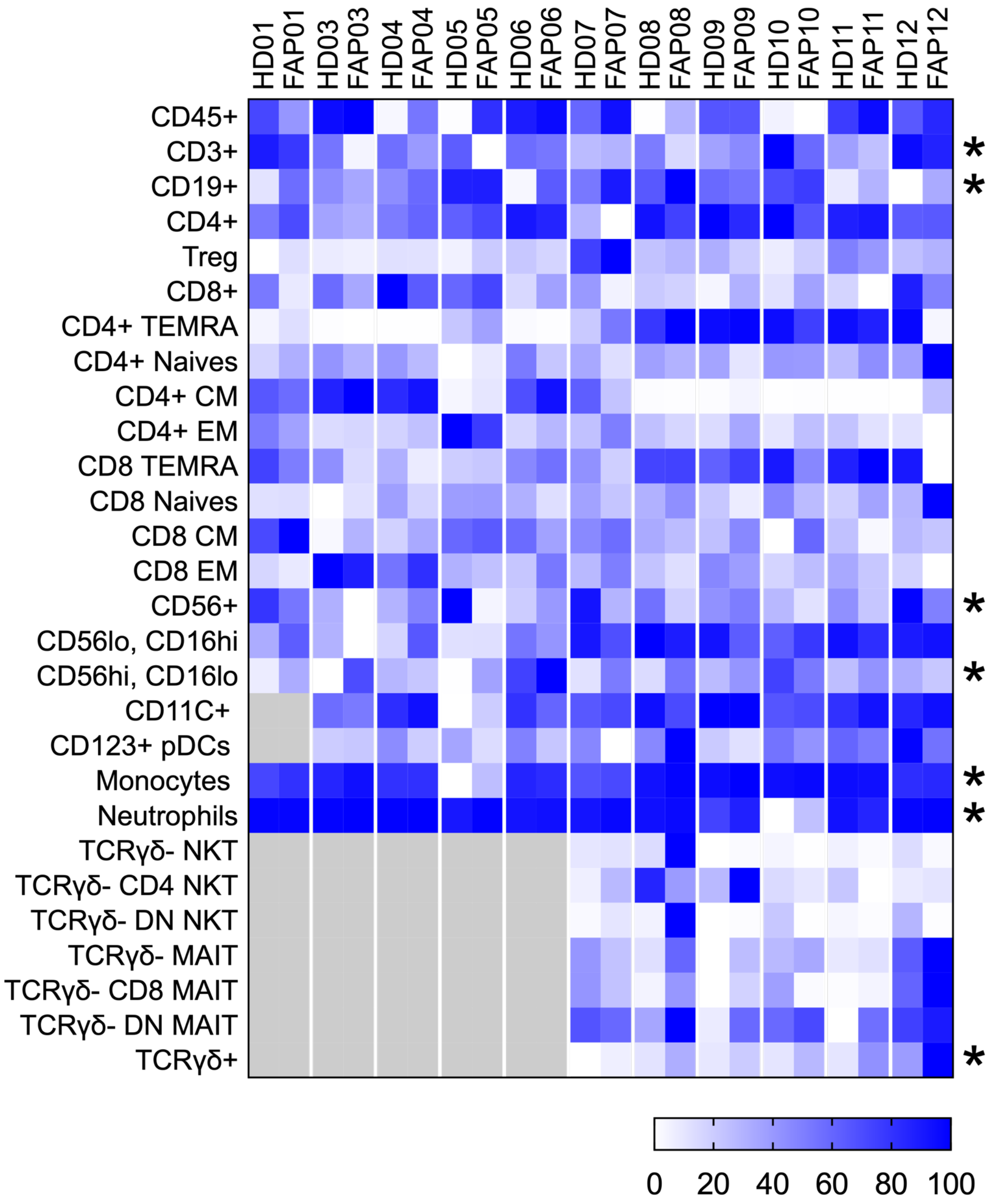

Supplementary Figure 6. **Whole blood immunophenotyping by multiparameter flow cytometry.** Whole blood was prepared, and immune cells were stained with directly-coupled fluorescent antibodies as detailed in materials and methods and in supplemental Table 1. Cells were analyzed by multiparameter flow cytometry. Gating strategies described and cell surface molecules defining cell populations are depicted in Supplemental Figure 1 and include lymphocytes, monocytes (Mono), dendritic cells (DC), neutrophils (Neutro), CD4 and CD8 T cells, NK, NKT, MAIT, Tγδ cells. The percentage of cells expressing the indicated markers for pairs of sex and age matched FAP and healthy subjects (n = 11, see Figure 3) is shown. Data were normalized and color-coded by setting the minimum value in each row at 0% (white) and the maximum value at 100% (dark blue). Gray squares indicate missing samples. Asterisks indicate markers with a significant difference between HD and FAP groups (see Figure 3; *p<0.05).

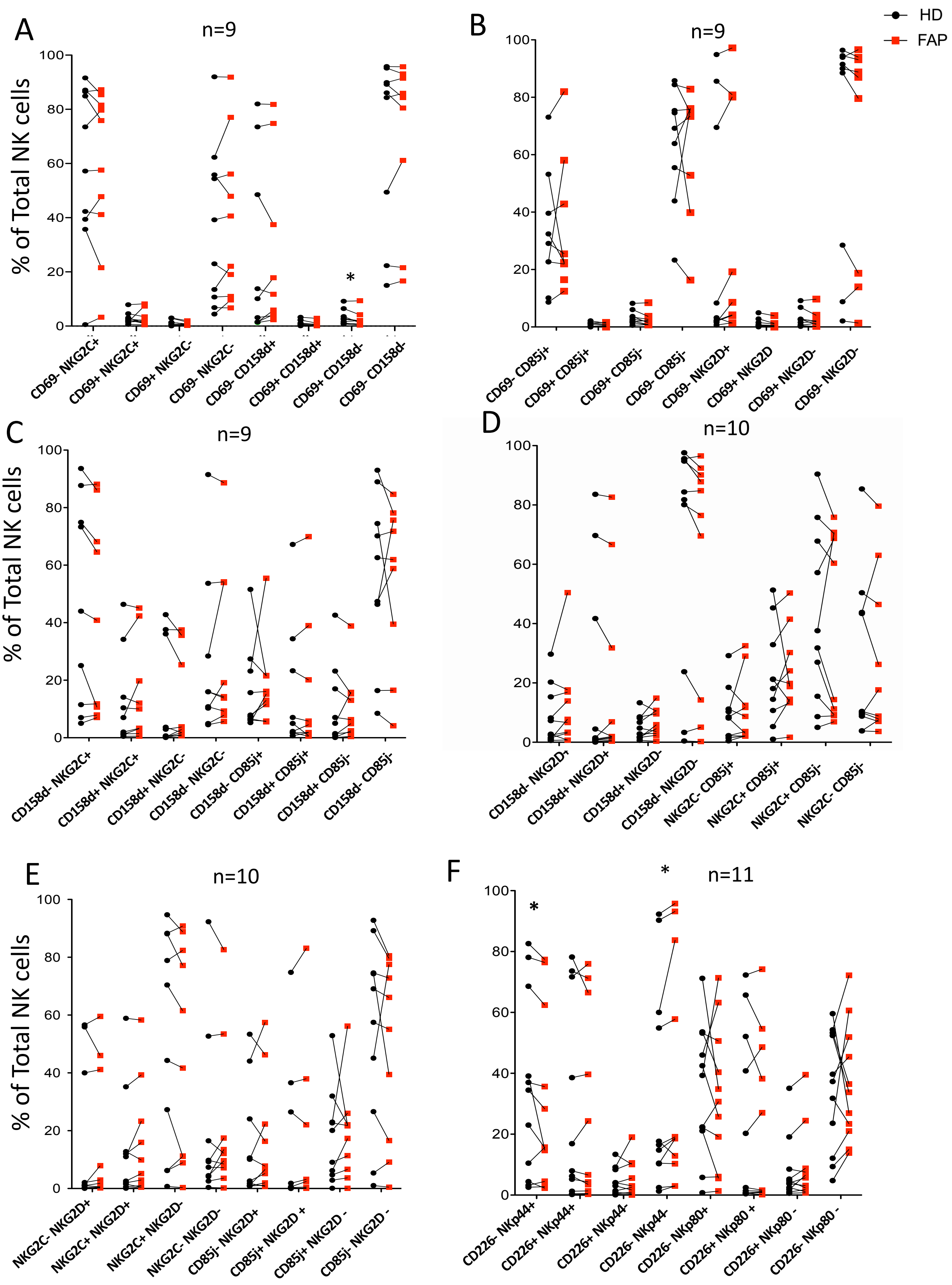

Supplementary Figure 7. Whole blood immunophenotyping of NK cell subpopulations

(Legend as in Supplementary Figure 9)

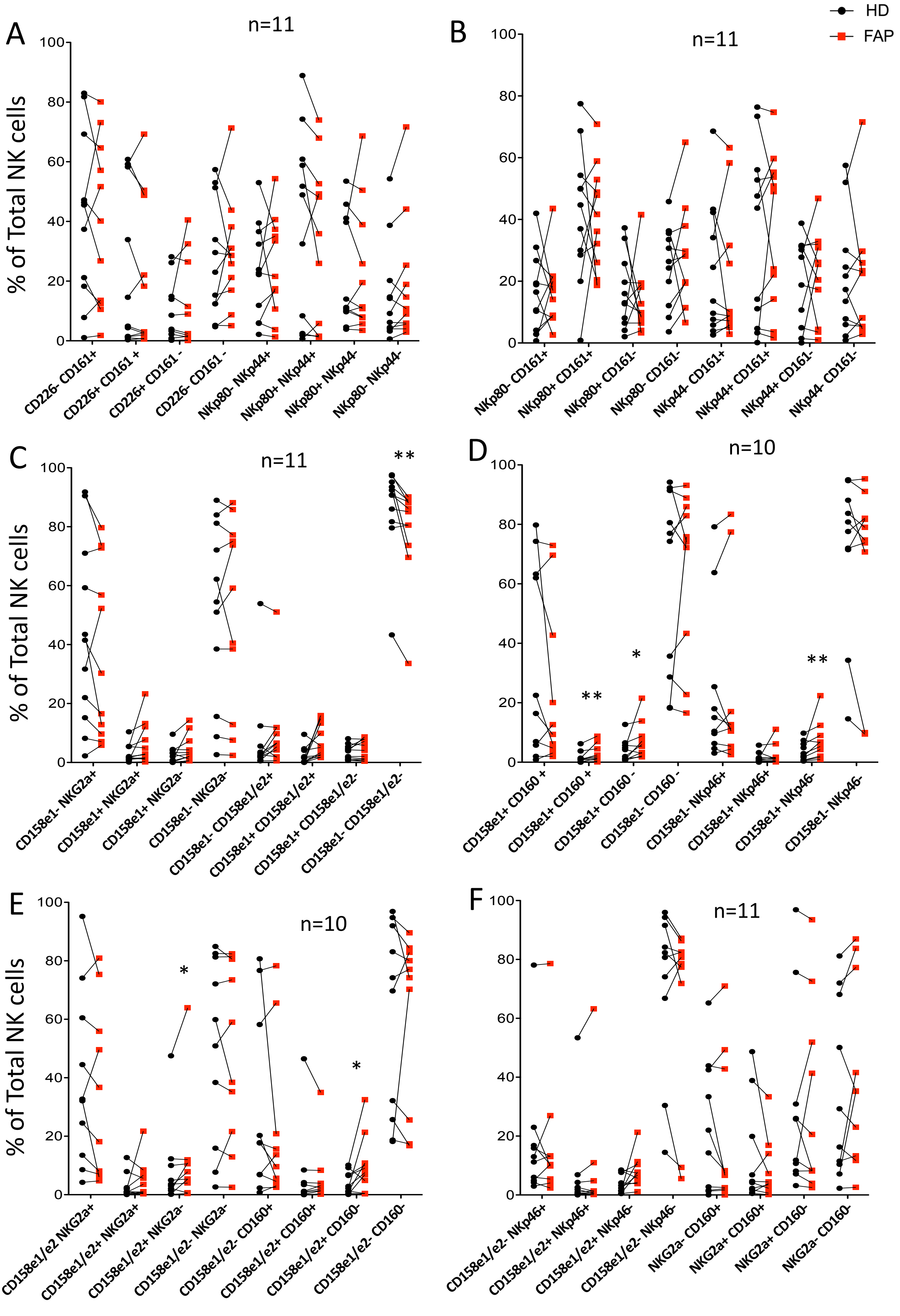

Supplementary Figure 8. **Whole blood immunophenotyping of NK cell subpopulations**
(Legend as in Supplementary Figure 9)

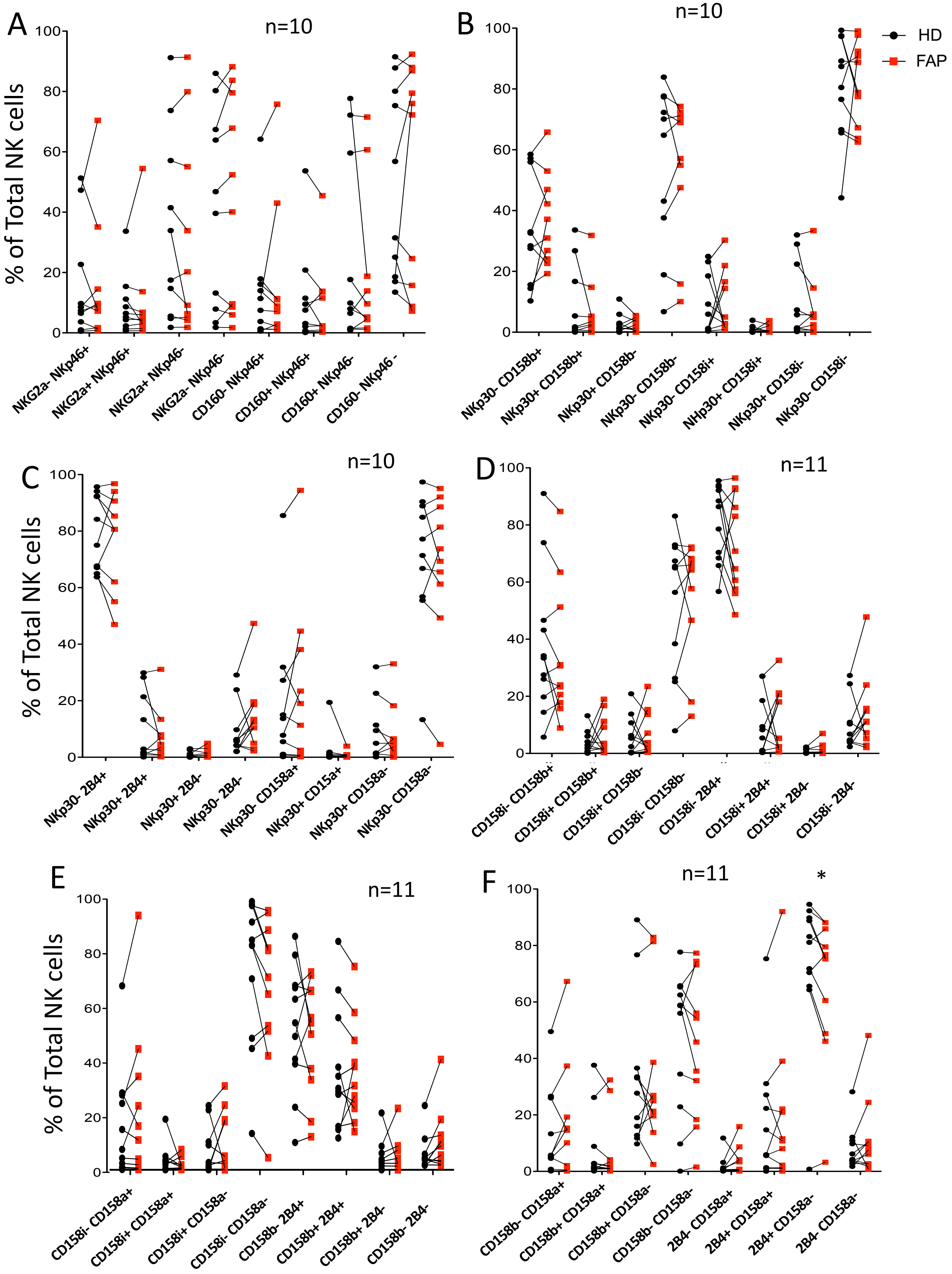

Supplementary Figure 9. **Whole blood immunophenotyping of NK cell subpopulations.**
Cells were immunophenotyped following the FACS gating strategy in Figure 1C. Each pair of sex and age matched FAP (red square dots) and healthy (black round dots) subjects is linked by a line. Each dot represents a subject. Percentage of cells expressing the characteristic markers is plotted. Significance was determined by the Wilcoxon rank test. ***p* < 0.01, **p* < 0.05. Significant differences are summarized in Table 1.

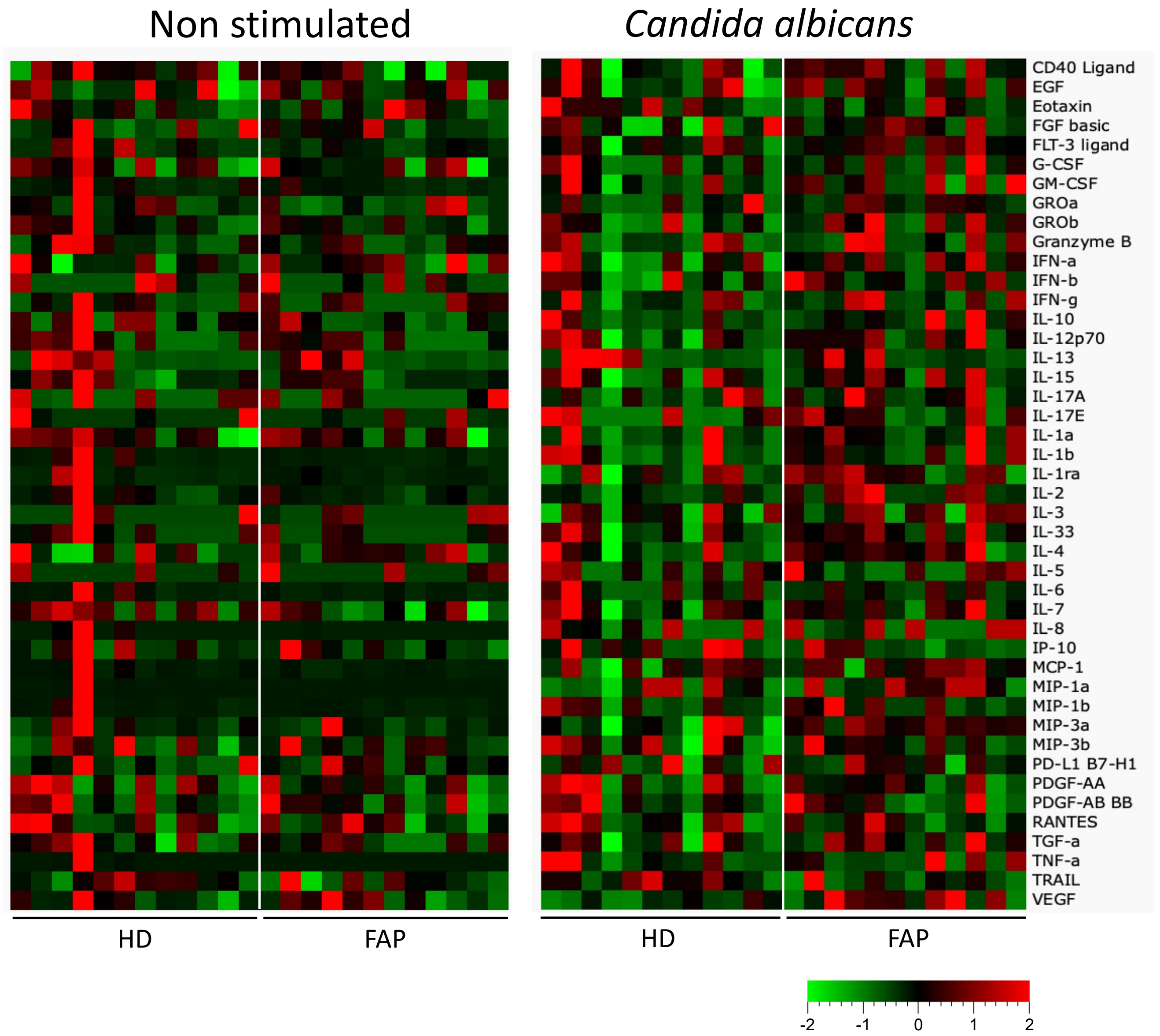

Supplementary Figure 10. Protein secretion pattern in whole blood.
(Figure legend as in Supplementary Figure 11)

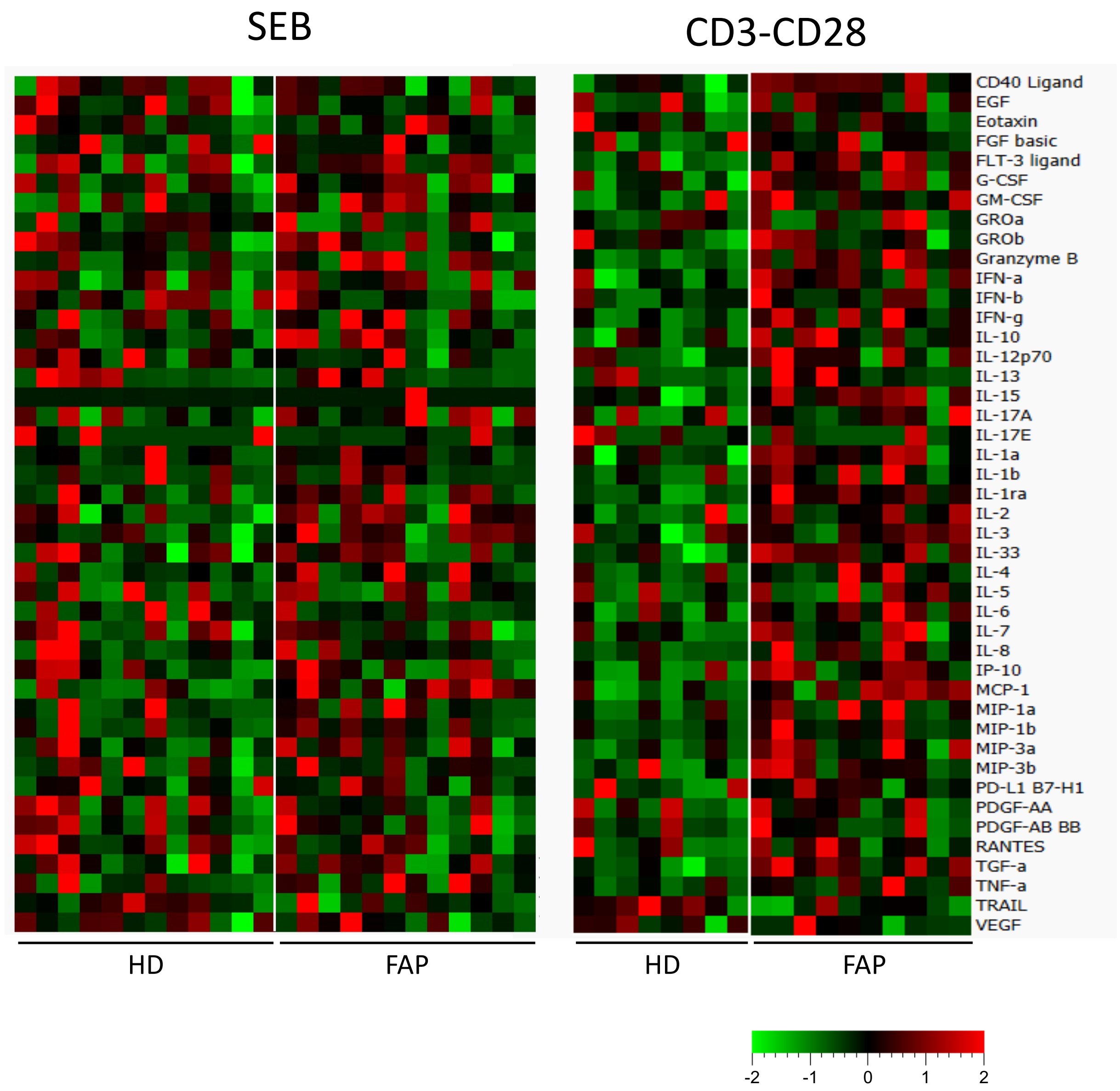

Supplementary Figure 11. Protein secretion pattern in whole blood. Whole blood from pairs of sex and age matched FAP and healthy subjects (HD) (one pair per incubation) was set into TruCulture tubes with no stimulus, *Candida albicans*, SEB, or anti-CD3+anti-CD28 stimuli and incubated during 22 hours at 37°C. Cells and supernatants were separated and the latter were kept frozen. All 12 pairs of FAP and healthy subjects were then analyzed together by Luminex technology using a panel of 44 analytes (supplemental table 3, Y-axis). Heat maps show the results for all the analytes. Color scale from green (-2) to bright red (2). Note that sample of patients that that did not respond to anti-CD3+anti-CD28 stimulation are not included in this analysis.

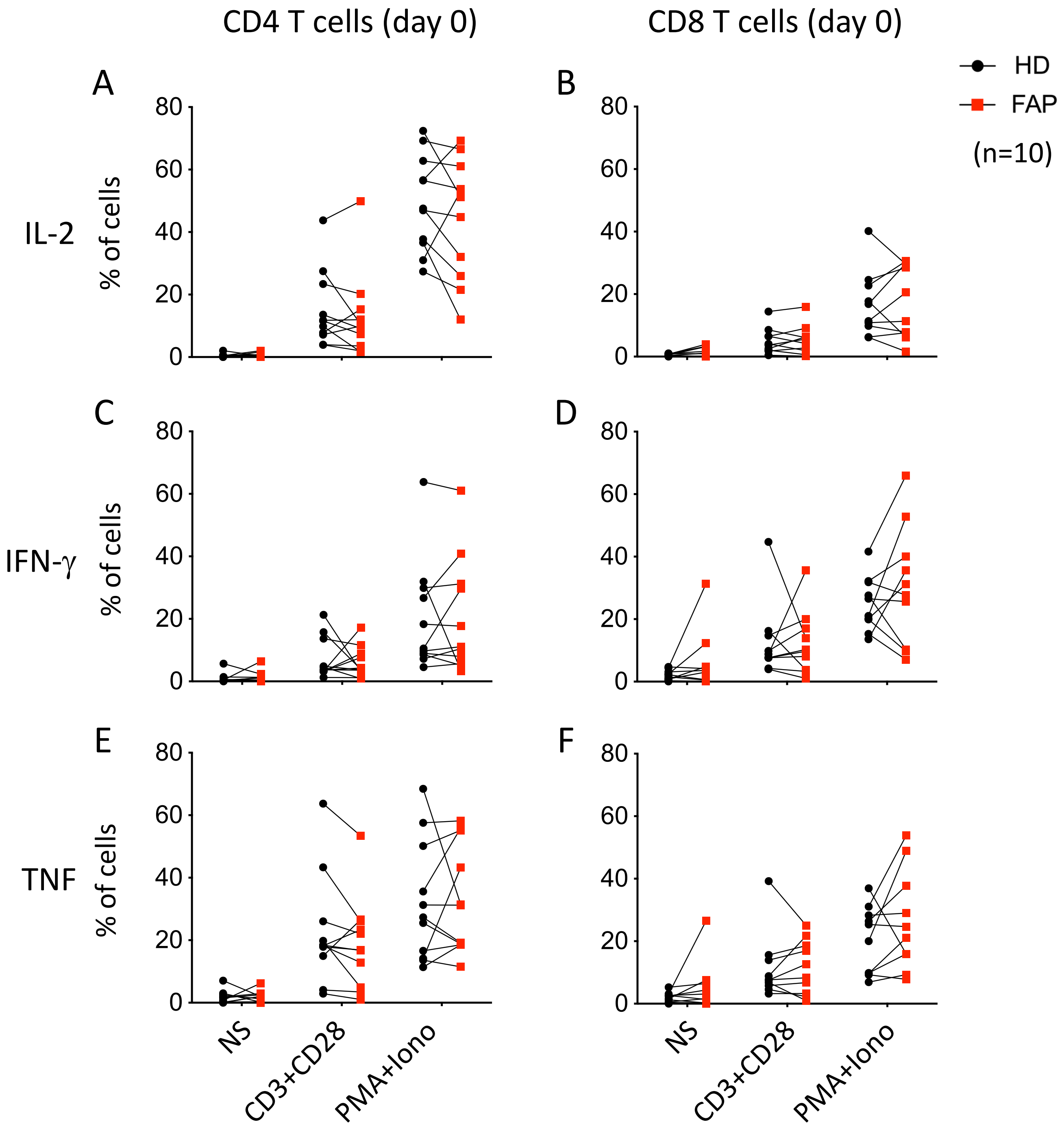

Supplementary Figure 12. Cytokine production by PBMC (day 0). Freshly isolated PBMC, (day 0), were left unstimulated (NS) or stimulated for 6 h with anti-CD3+anti-CD28, or with PMA+ionomycin. $CD4^+$ and $CD8^+$ T cells expressing IL-2, IFNγ and TNFα were monitored by intracellular cytokine staining and flow cytometry. Plots show the percentage of CD4 and CD8 T cells expressing IL-2 **(A, B)**, IFNγ **(C, D)**, TNFα **(E, F).** Each pair of sex and age matched FAP (red, square dots) and healthy (round black dots) subjects is shown linked by a line. Each dot represents a subject. n = number of pairs. Significance was determined by the Wilcoxon rank test. No significant differences were found under any condition, $p \geq 0.05$.

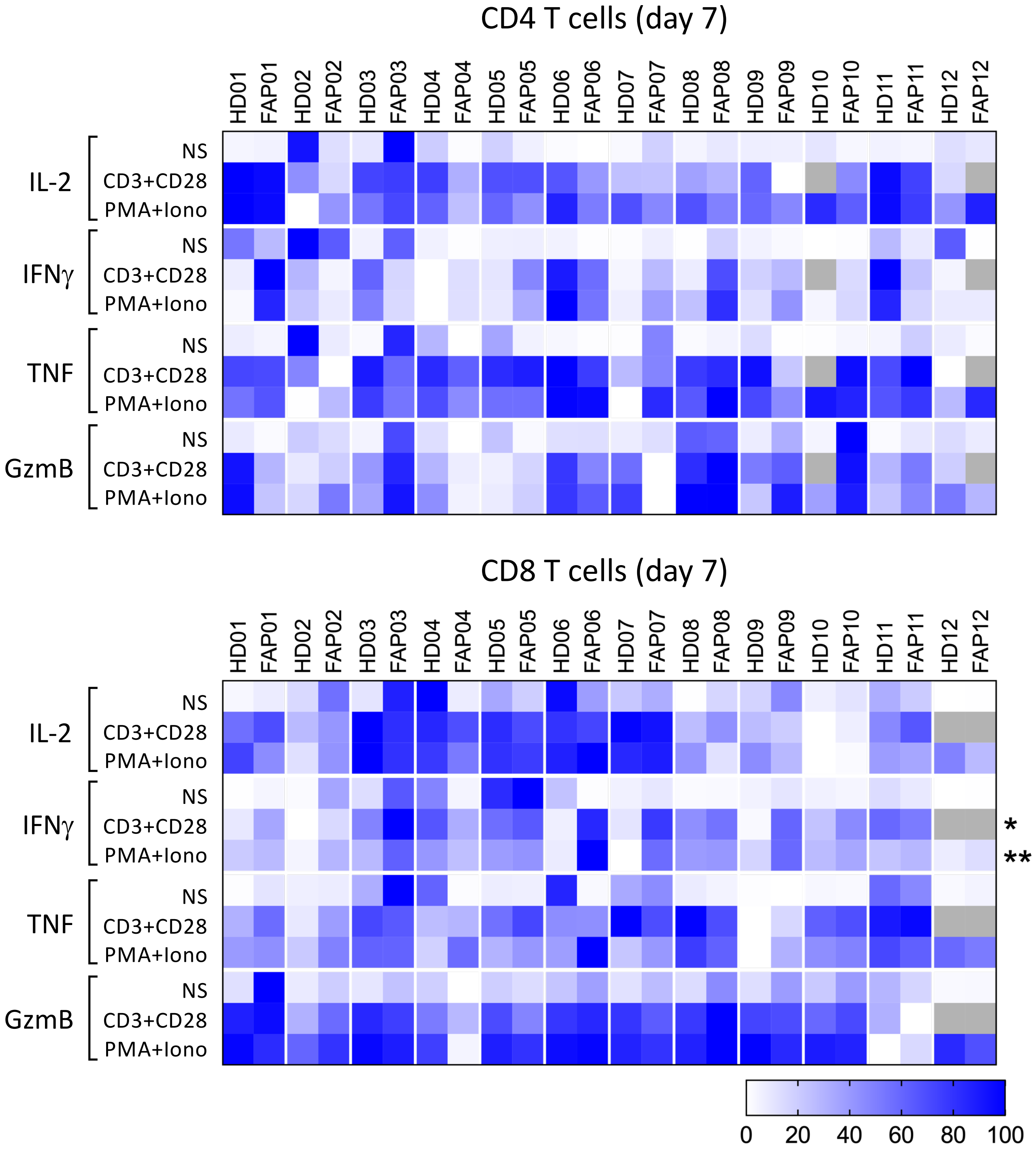

Supplementary Figure 13. Cytokine production by *in vitro*-expanded PBMC. T cells were expanded in vitro for 7 days by activation with anti-CD3+CD28 and cultured with IL-2. At day 7, cells were washed and restimulated for 6 h with anti-CD3+CD28 or with PMA+ionomycin. CD4+ and CD8+ T cells expressing IL-2, IFN-γ and TNF were monitored by intracellular cytokine staining and flow cytometry. In parallel, granzyme B expression was monitored. The percentage of CD4+ and CD8+ T cells expressing IL-2, IFN-γ, TNF and granzyme-B (GzmB) for pairs of sex and age matched FAP and healthy subjects (n = 12, see Figure 7) is shown. Data were normalized and color-coded by setting the minimum value in each row at 0% (white) and the maximum value at 100% (dark blue). Gray squares indicate missing samples. Asterisks indicate conditions with a significant difference between HD and FAP groups (determined as described in Figure 7; $^{**}p < 0.01$, $^{*}p < 0.05$).

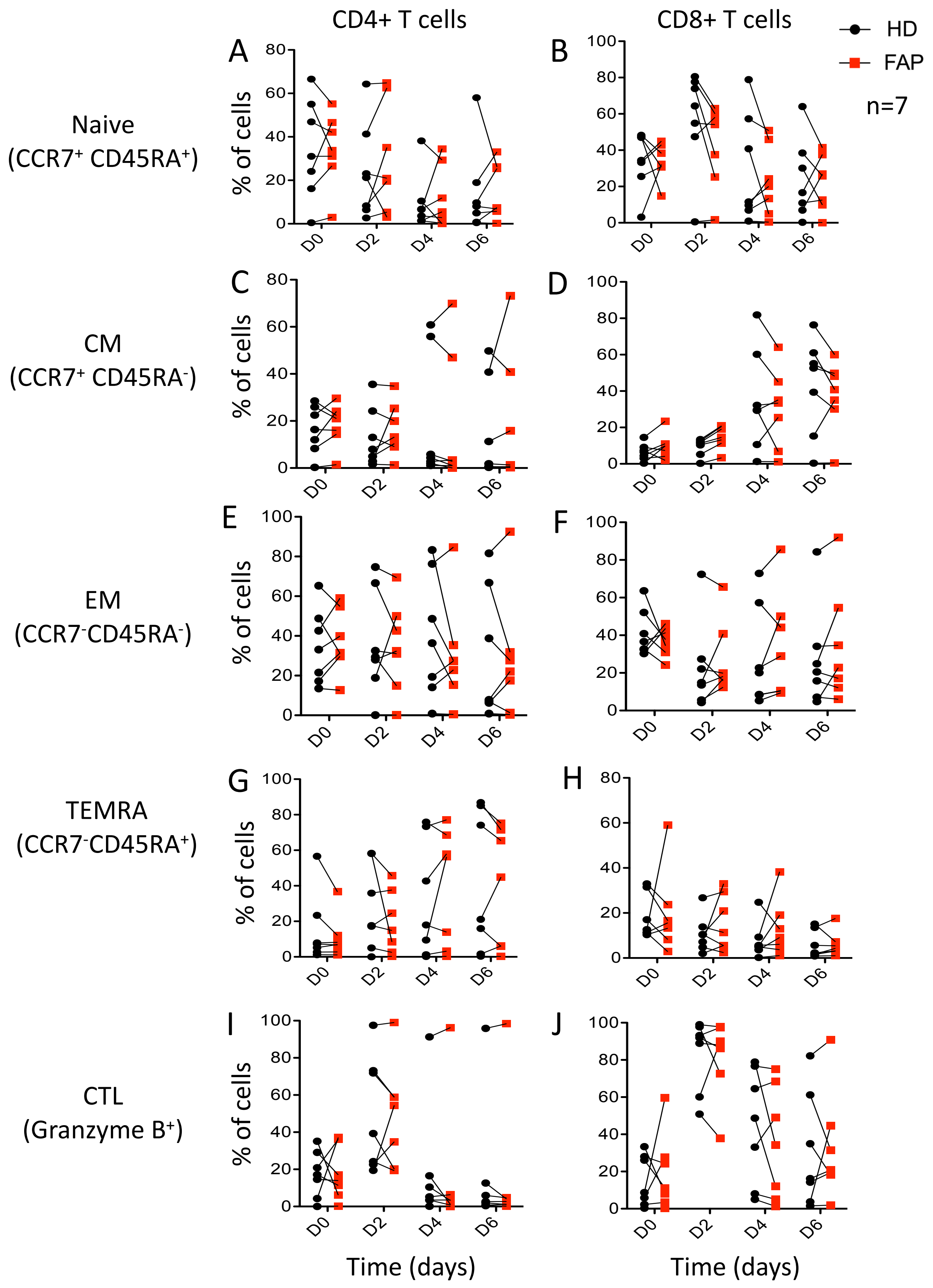

Supplementary Figure 14. ***In vitro*** **differentiation of PBMC.** Cells were stimulated *in vitro* with anti-CD3+CD28 Abs and IL-2 for 6 days. The expression of differentiation markers defining naïve, central memory (CM), effector memory (EM), terminally-differentiated effector cells (TEMRA) and CTL, was monitored at the indicated time points for CD4+ and CD8+ T cells, by FACS. Pairs of sex and age matched FAP (red, square dots) and healthy (black, round dots) subjects are shown linked by a line. Each dot represents a subject. Significance was determined by the Wilcoxon rank test, n=7 pairs. No significant differences were found ($p>0.05$).
